# CsiR-Mediated Signal Transduction Pathway in Response to Low Iron Conditions Promotes *Escherichia coli* K1 Invasion and Penetration of the Blood-Brain Barrier

**DOI:** 10.1093/infdis/jiae157

**Published:** 2024-03-26

**Authors:** Yangyang Zheng, Hao Sun, Yanling Wang, Chen Jin, Xiaoya Li, Yu Pang, Qianwen Ge, Lei Wang, Bin Liu

**Affiliations:** National Key Laboratory of Intelligent Tracking and Forecasting for Infectious Diseases, TEDA Institute of Biological Sciences and Biotechnology, Nankai University,Tianjin, People's Republic of China; Key Laboratory of Molecular Microbiology and Technology, Ministry of Education, Tianjin, People's Republic of China; Department of Microbiology, College of Life Sciences, Nankai University, Tianjin, People's Republic of China; National Key Laboratory of Intelligent Tracking and Forecasting for Infectious Diseases, TEDA Institute of Biological Sciences and Biotechnology, Nankai University,Tianjin, People's Republic of China; Key Laboratory of Molecular Microbiology and Technology, Ministry of Education, Tianjin, People's Republic of China; National Key Laboratory of Intelligent Tracking and Forecasting for Infectious Diseases, TEDA Institute of Biological Sciences and Biotechnology, Nankai University,Tianjin, People's Republic of China; Key Laboratory of Molecular Microbiology and Technology, Ministry of Education, Tianjin, People's Republic of China; National Key Laboratory of Intelligent Tracking and Forecasting for Infectious Diseases, TEDA Institute of Biological Sciences and Biotechnology, Nankai University,Tianjin, People's Republic of China; Key Laboratory of Molecular Microbiology and Technology, Ministry of Education, Tianjin, People's Republic of China; National Key Laboratory of Intelligent Tracking and Forecasting for Infectious Diseases, TEDA Institute of Biological Sciences and Biotechnology, Nankai University,Tianjin, People's Republic of China; Key Laboratory of Molecular Microbiology and Technology, Ministry of Education, Tianjin, People's Republic of China; National Key Laboratory of Intelligent Tracking and Forecasting for Infectious Diseases, TEDA Institute of Biological Sciences and Biotechnology, Nankai University,Tianjin, People's Republic of China; Key Laboratory of Molecular Microbiology and Technology, Ministry of Education, Tianjin, People's Republic of China; National Key Laboratory of Intelligent Tracking and Forecasting for Infectious Diseases, TEDA Institute of Biological Sciences and Biotechnology, Nankai University,Tianjin, People's Republic of China; Key Laboratory of Molecular Microbiology and Technology, Ministry of Education, Tianjin, People's Republic of China; National Key Laboratory of Intelligent Tracking and Forecasting for Infectious Diseases, TEDA Institute of Biological Sciences and Biotechnology, Nankai University,Tianjin, People's Republic of China; Key Laboratory of Molecular Microbiology and Technology, Ministry of Education, Tianjin, People's Republic of China; National Key Laboratory of Intelligent Tracking and Forecasting for Infectious Diseases, TEDA Institute of Biological Sciences and Biotechnology, Nankai University,Tianjin, People's Republic of China; Key Laboratory of Molecular Microbiology and Technology, Ministry of Education, Tianjin, People's Republic of China; Nankai International Advanced Research Institute, Shenzhen, People's Republic of China

**Keywords:** meningitis, *Escherichia coli* K1, transcriptional regulator, invasion, BBB penetration

## Abstract

*Escherichia coli* K1 is the leading cause of neonatal gram-negative bacterial meningitis, but the pathogenesis of *E coli* K1 meningitis remains unclear. Blood-brain barrier (BBB) penetration is a crucial step in *E coli* meningitis development. Here, we uncovered the crucial role of CsiR, a GntR family regulator, in *E coli* K1 virulence. During infection, *csiR* expression was induced due to the derepression by Fur in the blood and human brain microvascular endothelial cells (HBMECs). CsiR positively regulated *ilvB* expression, which is associated with branched chain amino acid synthesis. Furthermore, we revealed that IlvB activated the FAK/PI3K pathway of HBMECs to induce actin cytoskeleton rearrangements, thereby promoting the bacterial invasion and penetration of the BBB. Overall, this study reveals a CsiR-mediated virulence regulation pathway in *E coli* K1, which may provide a useful target for the prevention or therapy of *E coli* meningitis.

## Teaser

Our work uncovers a new CsiR-mediated virulence regulatory pathway that enhances the ability of *Escherichia coli* K1 to invade host cells and penetrate the blood-brain barrier, which may provide a useful target for the prevention or therapy of *E coli* meningitis.

Central nervous system (CNS) infection remains a major cause of high mortality and morbidity [1]. Bacterial meningitis, a meningeal inflammation that affects the pia mater, arachnoid, and subarachnoid space, ranks among the top 10 causes of infection-related deaths globally [2]. *Escherichia coli*, with the K1 capsular polysaccharide predominating, has become the most prevalent gram-negative pathogen of early-onset sepsis and bacterial meningitis in newborns [3]. Upon entering the intravascular space, *E coli* K1 first survives and proliferates within the blood, resulting in bacteremia, a prerequisite to penetrating the blood-brain barrier (BBB). The human BBB, which primarily comprises a single layer of specialized human brain microvascular endothelial cells (HBMECs), serves as a critical barrier against microbial invasion of the CNS [4]. *Escherichia coli* K1 can penetrate the BBB through the transcellular pathway, including microbial adhesion, invasion, and intracellular traversal [5]. Several *E coli*–assisted HBMEC-penetrating determinants, including IbeA, OmpA, and CNF1, have been implicated in host cell actin cytoskeleton rearrangement via the activation of focal adhesion kinase (FAK), phosphatidylinositol 3-kinase (PI3K), Rac1, or RhoA signaling [6–10]. As many unknown factors may be associated with bacterial invasion of HBMECs, identifying these factors and analyzing the mechanisms underlying their promotion of bacterial invasion could help uncover the pathogenesis of *E coli* K1 meningitis.

Iron serves as a vital cofactor for many enzymes in bacteria, playing a crucial role in diverse physiological processes, including respiration, tricarboxylic acid cycling, oxidative stress resistance, and DNA synthesis [11–14]. While iron is a naturally abundant nutrient, the amount of free iron in the blood is extremely minimal because iron is bound to storage molecules such as ferritin and hemosiderin [15]. After infecting the host, the bacteria must inevitably compete with the host for iron. Some bacteria even exploit iron as a signal to promote their own virulence [16].

To survive and thrive in the blood and HBMECs, *E coli* K1 monitors its surroundings while adjusting its gene expression and physiology accordingly, which largely depends on transcriptional regulators, 2-component systems, and regulatory RNAs. The GntR family remains a predominant group of helix-turn-helix bacterial metabolite-responsive transcriptional factors [17]. Many GntR family transcriptional regulators have been characterized as controlling numerous cellular processes, such as motility, glucose metabolism, bacterial resistance, pathogenesis, and virulence [18]. However, the role of GntR family regulators in the virulence regulation of *E coli* K1 remains underexplored.

In this study, we characterized a GntR family regulator, CsiR, and demonstrated its significant role in *E coli* K1 virulence. During infection, *csiR* expression was upregulated due to the derepression of Fur through its “Fur box” of the promoter in response to low iron conditions in the blood and HBMECs. CsiR contributed to bacterial virulence by directly binding to the promoter of *ilvB* and activating its transcription, which is associated with branched chain amino acid (BCAA) synthesis. Moreover, we found that *ilvB* promoted bacterial internalization in HBMECs by inducing actin cytoskeleton rearrangements through the FAK/PI3K signaling pathway. Overall, this study uncovers a new CsiR-mediated regulatory pathway that enhances the ability of *E coli* K1 to invade host cells and penetrate the BBB.

## METHODS

### Bacterial Strains, Plasmids, and Growth Conditions

The *E coli* strain RS218 (O18:K1:H7) with a K1 capsule, a prototype meningitis isolated from the cerebrospinal fluid of a newborn infant [19], was designated as wild-type (WT) and used throughout the study. The bacterial strains, plasmids, and primers used in this study are listed in Supplementary Tables 1 and 2. The mutant strains were generated using the λ Red recombinase system [20]. To create complemented strains, the amplification products containing the open reading frame and upstream promoter sequence of the respective genes were cloned into the pTrc99a plasmid. Subsequently, the recombinant plasmids were transformed into mutant strains. Fur-ON mutant strain (Fur H118A) was constructed by substituting H118 to A118 of Fur in the genome of RS218 [21]. All the strains were cultured in Luria-Bertani (LB) broth or agar at 37°C. Ampicillin, chloramphenicol, or kanamycin was added at 100, 25, or 50 μg/mL, respectively, as appropriate.

### Statistical Analysis

All data in this study are representative of 3 separate experiments unless otherwise stated and displayed as mean ± standard deviation. Statistical analysis was performed using GraphPad Prism version 9.3.1 software. Differences between 2 groups were evaluated using 2-tailed unpaired Student *t* test or 2-way analysis of variance (ANOVA), and differences in 3 or more groups were evaluated using 1-way ANOVA according to the test requirements. Significance is indicated as the *P* value.

Detailed experimental protocols, materials, and methods are described in the Supplementary Methods.

## RESULTS

### *csiR* Mutant Exhibits Reduced Efficiency in Invading and Traversing HBMECs

**Figure 1.**
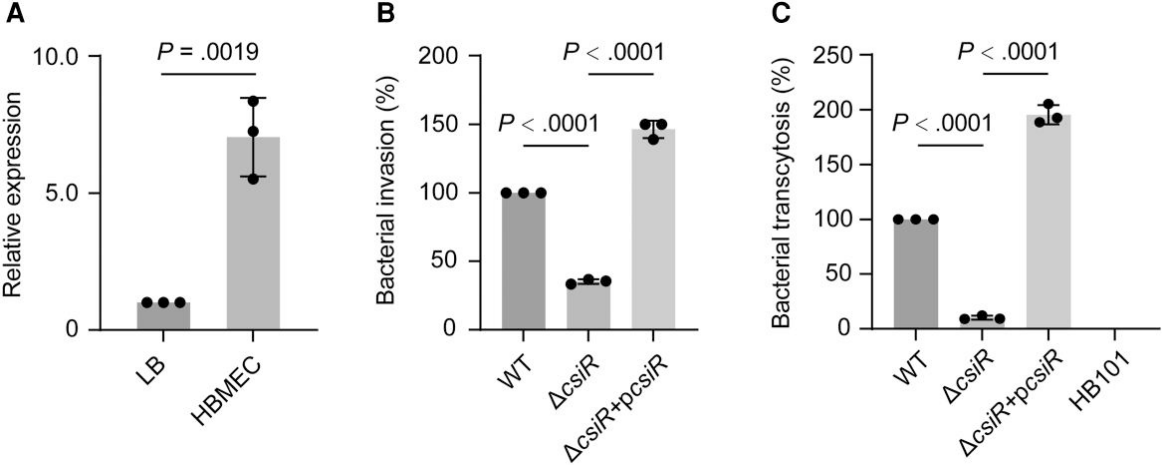
CsiR promotes *Escherichia coli* K1 invasion and transcytosis of human brain microvascular endothelial cells (HBMECs). *A*, Quantitative reverse-transcription polymerase chain reaction expression level of *csiR* of wild type (WT) in Luria-Bertani (LB) medium and the host HBMEC. The 2^−ΔΔCt^ method was employed to calculate the fold change in the target gene relative to the housekeeping gene (16S rRNA). *B*, Differences in the invasion of the Δ*csiR* and complemented strain relative to that of WT. *C*, Differences in the transcytosis of the Δ*csiR* and complemented strain relative to that of WT. Nonpathogenic strain HB101, which cannot penetrate the blood-brain barrier, serves as a negative control. Data are presented as mean ± standard deviation (n = 3). Significance is indicated by the *P* values calculated using Student *t* test.

Penetration of the brain by *E coli* K1 involves its binding to and invasion of HBMECs [22]. Analysis of published transcriptome data revealed that the expression of *csiR*, which encodes a GntR superfamily protein of transcriptional regulators [23], was upregulated during bacterial infection of HBMECs. To verify the RNA-seq data, the HBMECs were infected with a WT strain. The quantitative reverse-transcription polymerase chain reaction (qRT-PCR) results revealed that *csiR* expression in HBMECs was 7.04-fold higher than its expression in LB broth (Figure 1*A*).

To investigate the effect of *csiR* on *E coli* K1 pathogenicity, WT, Δ*csiR*, and a complemented strain (Δ*csiR* + p*csiR*) were used to infect HBMECs. The results showed that the invasion and the transcytosis rates of Δ*csiR* exhibited a 2.85-fold and 4.68-fold decrease compared to WT, respectively (Figure 1*B* and 1*C*). The Δ*csiR* grew as well as WT in both LB broth and Dulbecco’s modified Eagle medium (DMEM), respectively (Supplementary Figure 1*A* and 1*B*) and adhered HBMECs as efficiently as WT and the complemented strain (Supplementary Figure 1*C*), indicating that the reduced invasion and transcytosis ability of Δ*csiR* were not due to growth defects or a reduced adhesion ability.

### *csiR* Is Controlled by a Global Regulator *fur* in Response to Iron Availability

**Figure 2.**
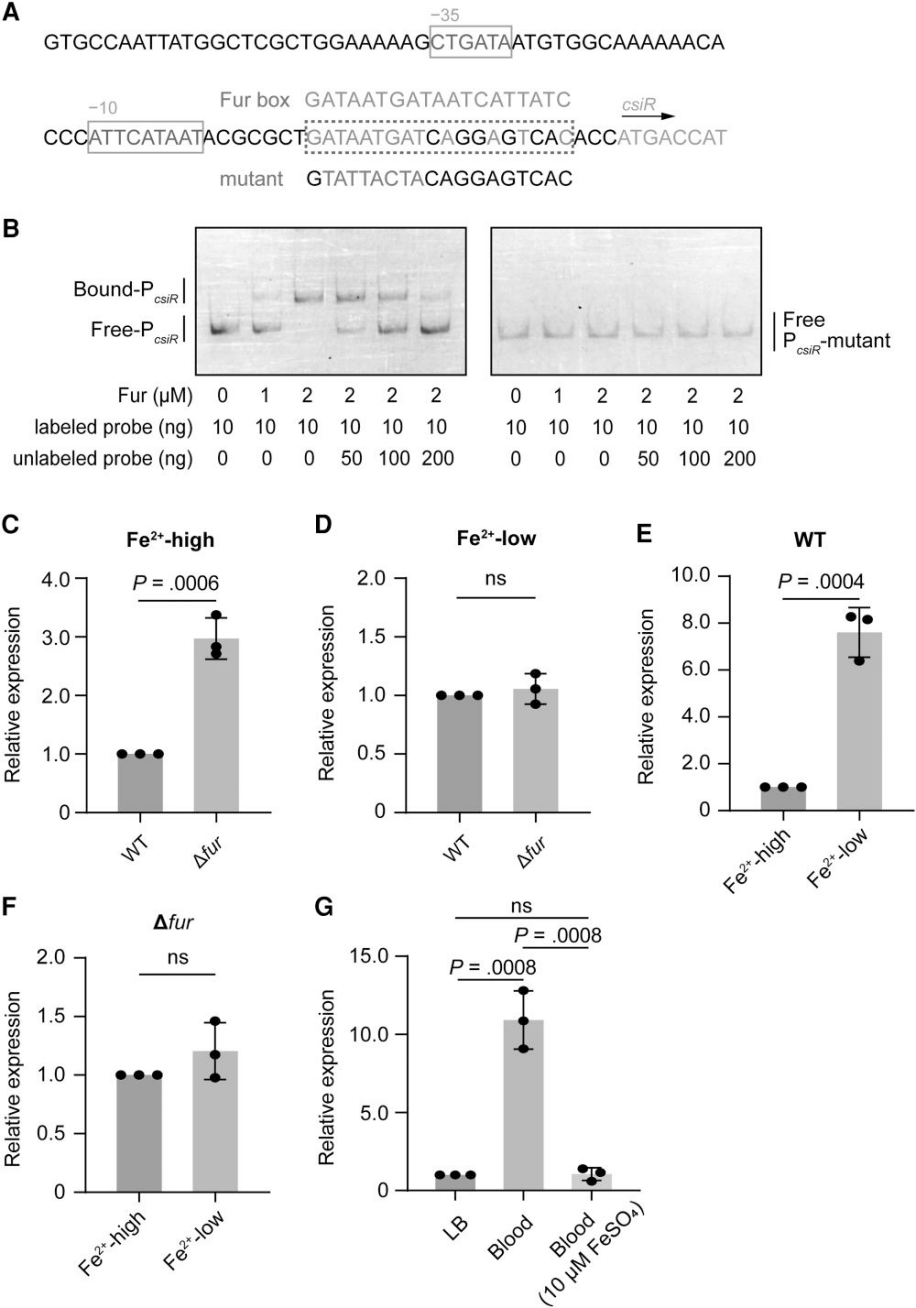
*csiR* is directly repressed by Fur in response to iron availability. *A*, Part of the sequence of the *csiR* promoter with −35 and −10 regions as well as the potential Fur box are marked by different boxes. Alignment of the consensus sequences, putative Fur box, and the mutant Fur box sequences in the *csiR* promoter. *B*, Electrophoretic mobility shift assay and competition assay of the specific binding of purified Fur to the putative Fur box and the mutant Fur box of *csiR*. *C* and *D*, Quantitative reverse-transcription polymerase chain reaction (qRT-PCR) expression level of *csiR* in wild type (WT) and Δ*fur* under iron-sufficient (Fe^2+^-high, *C*) or iron-deficient (Fe^2+^-low, *D*) condition. *E* and *F*, qRT-PCR expression level of *csiR* in WT (*E*) or Δ*fur* (*F*) under iron-sufficient (Fe^2+^-high) and iron-deficient (Fe^2+^-low) conditions, respectively. *G*, qRT-PCR expression level of *csiR* of WT in Luria-Bertani (LB) medium and blood with or without 10 μM ferrous sulfate (FeSO_4_) of mice receiving WT via the tail vein. Data are presented as mean ± standard deviation (n = 3). Significance is indicated by the *P* values calculated using Student *t* test; ns, not significant.

Because *csiR* was induced after *E coli* K1 infection of HBMEC, we investigated which regulator and signal inside HBMEC induced the CsiR regulatory system. We first predicted *csiR* characteristic regions using the online promoter prediction tool Softberry (http://linux1.softberry.com/berry.phtml?topic=index&group=programs&subgroup=promoter), and we were surprised to uncover a Fur box located near the −10 region of the *csiR* promoter (Figure 2*A*). Electrophoretic mobility shift assay (EMSA) and competition assay showed that purified Fur protein bound to the *csiR* promoter in vitro (Figure 2*B*, Supplementary Figure 2*A*). By contrast, when we mutated the conserved residues of the Fur box of the *csiR* promoter from ATAATGAT to TATTACTA or used a DNA fragment derived from the *kana* gene as the negative, Fur binding was eliminated (Figure 2*B*, Supplementary Figure 2*B*). These data indicate that Fur directly regulates *csiR* at a specific binding site of the Fur box.

Since the Fur protein forms a complex with ferrous iron and represses the expression of a subset of genes encoding iron uptake systems [24], we tested whether Fur regulates *csiR* expression. In Δ*fur*, the *csiR* expression was significantly increased compared with WT under the iron-sufficient condition by adding 100 μM ferrous sulfate (FeSO_4_) (Figure 2*C*) and comparable under the iron-deficient condition by adding 100 μM 2,2′-dipyridyl, the iron chelator (Figure 2*D*). In addition, evaluation of the regulation performance of *csiR* under the 2 conditions in WT or Δ*fur* revealed an obvious increased *csiR* expression of 7.60-fold under the iron-deficient condition in WT (Figure 2*E*), with an insignificant difference observed in Δ*fur* (Figure 2*F*). These results suggest that *csiR* expression is repressed by Fur under iron sufficiency and this repression is relieved under iron deficiency. Furthermore, we also tested the *csiR* expression in vivo. We performed qRT-PCR analysis in the mouse blood supplemented with or without FeSO_4_. The result showed that *csiR* expression was significantly decreased in the blood supplemented with 10 μM FeSO_4_ (iron-sufficient condition), indicating the *csiR* is indeed induced in the lack of iron in vivo (Figure 2*G*).

We constructed a Fur H118A mutant strain that disturbs the UMPylation and therefore inactivated the derepression of Fur [21]. As a result, Fur H118A mutant strain continues to bind on *csiR* promoter even in the iron-deficient conditions and serves as a Fur-ON strain. The Δ*fur* and Fur H118A exhibited similar growth rates to WT in vitro (Supplementary Figure 2*C* and 2*D*). We then infected HBMECs with WT, Δ*fur*, and Fur H118A, and found that the adhesion rate were comparable with WT in either Δ*fur* or Fur H118A (Supplementary Figure 2*E* and 2*G*). Compared with WT, the invasion and transcytosis rates observed in Δ*fur* were not significantly different (Supplementary Figure 2*E*), consistent with the expression of *csiR* in Δ*fur* under the iron-deficient condition (Figure 2*D*, Supplementary Figure 2*F*). However, the invasion and transcytosis rates were significantly decreased in Fur H118A (Supplementary Figure 2*G*), suggesting that *csiR* expression is directly repressed by Fur through the binding site of the Fur box in the *csiR* promoter under iron-sufficient conditions, but when *E coli* K1 is in the blood of mice or HBMECs, which are iron-deficient conditions, *csiR* expression is activated due to derepression by Fur.

### *csiR* Regulates *ilvB* Expression Fur-Dependently Upon Sensing Iron Concentrations

**Figure 3.**
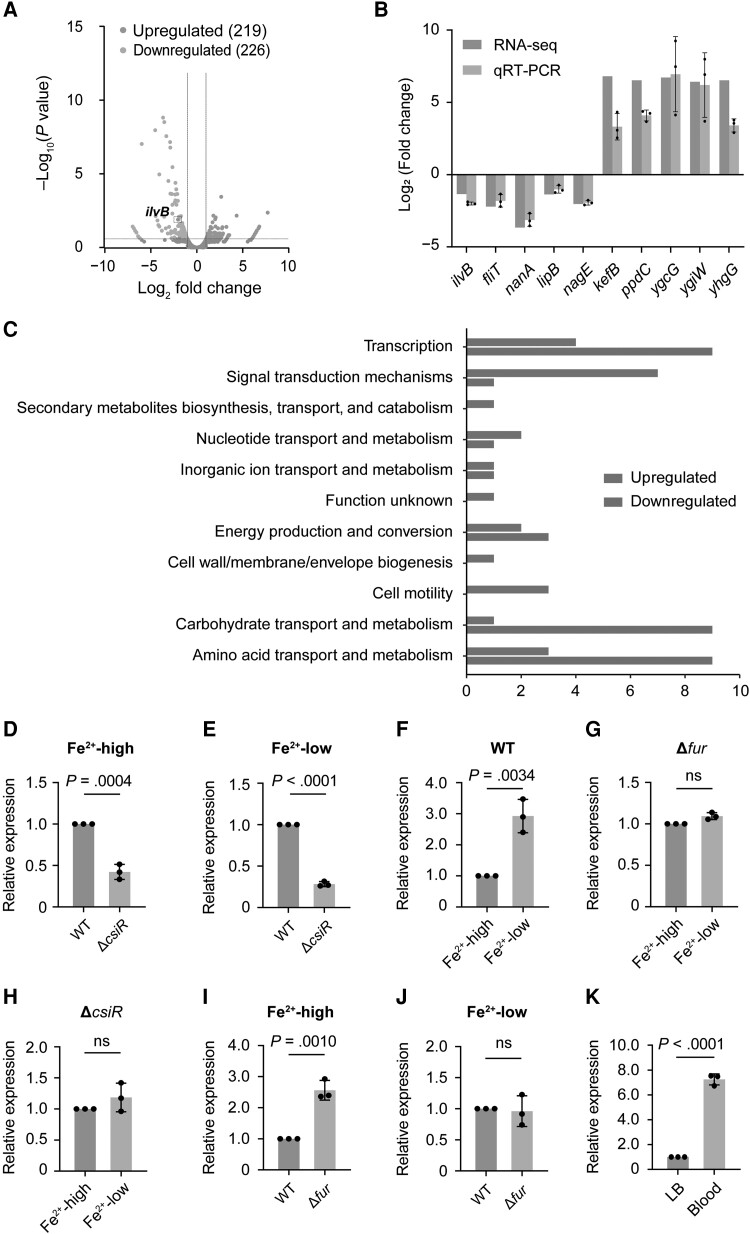
*csiR* promotes *ilvB* expression in a Fur-dependent manner by sensing iron concentration. *A*, The volcano plot for differential genes in wild type (WT) and Δ*csiR* that infected human brain microvascular endothelial cells. *B*, RNA-seq result validation by quantitative reverse-transcription polymerase chain reaction (qRT-PCR). *C*, Clusters of orthologous group analysis of the differentially regulated genes of Δ*csiR*. Bars represent the number of upregulated (light red) or downregulated (light blue) genes. *D* and *E*, qRT-PCR expression level of *ilvB* in WT and Δ*csiR* under iron-sufficient (Fe^2+^-high, *D*) or iron-deficient (Fe^2+^-low, *E*) condition. *F–H*, qRT-PCR expression level of *ilvB* in WT (*F*), Δ*fur* (*G*), or Δ*csiR* (*H*) under iron-sufficient (Fe^2+^-high) and iron-deficient (Fe^2+^-low) conditions. *I* and *J*, qRT-PCR expression level of *ilvB* in WT and Δ*fur* under iron-sufficient (Fe^2+^-high, *I*) or iron-deficient (Fe^2+^-low, *J*) condition. *K*, qRT-PCR expression level of *ilvB* of WT in Luria-Bertani medium and blood of mice receiving WT via the tail vein. Data are presented as mean ± standard deviation (n = 3). Significance is indicated by the *P* values calculated using Student *t* test; ns, not significant.

To analyze the mechanism by which *csiR* regulates the invasion and transcytosis of *E coli* K1, RNA-seq of WT and Δ*csiR* strains that infected HBMECs was performed. A total of 445 genes exhibited differential expression between WT and Δ*csiR*, with 219 upregulated and 226 downregulated in the Δ*csiR* (Figure 3*A*, Supplementary Table 3); the heatmap of the genes statistically significantly different in the RNA-seq data is presented in Supplementary Figure 3*A*. More than 20 of the differentially expressed genes, including the most significantly up- and downregulated genes, were selected for qRT-PCR analysis to validate the RNA-seq data. The results of qPCR strongly correlated with the transcriptome data, suggesting that RNA-seq–identified transcriptional changes were reliable (Figure 3*B*, Supplementary Figure 3*B*).

Genes that exhibited altered transcription levels were classified using the National Center for Biotechnology Information cluster of the orthologous group (COG) functional category annotation system. The COG categories enriched in the upregulated gene group were primarily involved in signal transduction mechanisms and cell motility (Figure 3*C*). The COG categories enriched in the list of downregulated genes included transcription; carbohydrate transport and metabolism; and amino acid transport and metabolism (Figure 3*C*).

According to a comprehensive analysis of the transcriptome data and the COG result, we found that the expression of *ilvB*, which encodes the structural gene for the 3 acetohydroxy acid synthase isozymes that catalyze the first common step in the biosynthesis of isoleucine, valine, and leucine in *E coli* K-12 [19], was significantly downregulated in Δ*csiR*. To verify whether CsiR and iron concentration regulate *ilvB* expression, we performed qRT-PCR assays to analyze *ilvB* expression in WT, Δ*fur*, and Δ*csiR* under iron-sufficient and -deficient conditions, respectively. The results showed that *ilvB* expression decreased by 2.37-fold and 3.53-fold in Δ*csiR* compared to WT under iron-sufficient and -deficient conditions, respectively, suggesting that CsiR positively regulates *ilvB* (Figure 3*D* and 3*E*). In addition, *ilvB* expression was increased by 2.92-fold under the iron-deficient condition compared with that under the iron-sufficient condition in WT (Figure 3*F*), with no significant difference observed under the same conditions in both Δ*fur* (Figure 3*G*) and Δ*csiR* (Figure 3*H*). These results demonstrate that iron-deficient conditions induce *ilvB* expression through Fur and CsiR activity. We also found that in an iron-sufficient environment where Fur represses *csiR* expression, *ilvB* expression increased 2.56-fold in Δ*fur* (Figure 3*I*), with no significant difference observed in an iron-deficient environment (Figure 3*J*), corroborating *csiR* expression results under the same condition (Figure 2*C* and 2*D*). Evaluation of *ilvB* expression in mouse blood, considering the low iron concentration of the blood, revealed a significant increase in *ilvB* expression compared to LB broth (Figure 3*K*). These results indicate the *ilvB* expression is Fur-dependently and positively regulated by CsiR in response to iron-deficient conditions.

### *csiR* Increases Bacterial BBB Penetration by Enhancing *ilvB* Expression Through Direct Binding to Its Promoter Region

**Figure 4.**
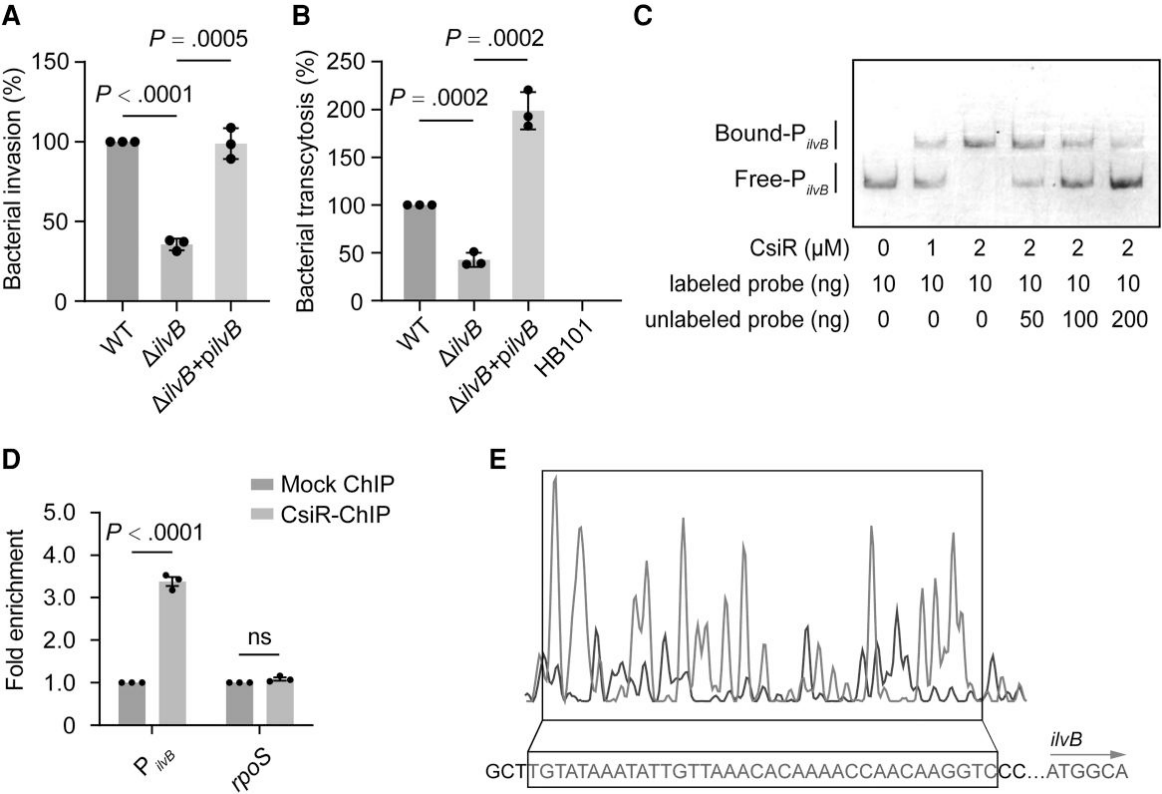
*csiR* directly increases the expression of *ilvB*. *A*, Differences in the invasion of the Δ*ilvB* and complemented strain relative to that of the wild-type (WT) strain. *B*, Differences in the transcytosis of the Δ*ilvB* and complemented strain relative to that of WT. Nonpathogenic strain HB101, which cannot penetrate the blood-brain barrier, serves as a negative control. *C*, Electrophoretic mobility shift assay and competition assay of the specific binding of purified CsiR to the promoter region of *ilvB*. *D*, The fold enrichment of the promoters for *ilvB* and the negative control (*rpoS*) in the chromatin immunoprecipitation (ChIP) assay. *E*, CsiR binds to a motif in the *ilvB* promoter region. The protected region shows a significantly reduced peak intensity (blue) pattern compared to the intensity seen in the control (red). The identified CsiR-binding motif is shown in a box at the bottom of the figure. Data are presented as mean ± standard deviation (n = 3). Significance is indicated by the *P* values calculated using the Student *t* test (*A* and *B*) and 2-way analysis of variance (*D*); ns, not significant.

Here, invasion and transcytosis assays were conducted to investigate whether *ilvB* promotes bacterial virulence. The growth curve of Δ*ilvB* was the same as that of WT in both LB broth and DMEM, respectively (Supplementary Figure 4*A* and 4*B*). The HBMEC infection results showed that the invasion and transcytosis rates of Δ*ilvB* exhibited a 2.62-fold and 2.41-fold decrease compared to WT, respectively (Figure 4*A* and 4*B*), while no difference in bacterial adhesion was observed between Δ*ilvB* and WT (Supplementary Figure 4*C*). The defects of Δ*ilvB* in cell invasion and transcytosis ability were restored by the complement of *ilvB*, indicating that *ilvB* plays an important role in *E coli* K1 crossing the BBB.

EMSA and competition assays showed that purified CsiR protein bound to the promoter of *ilvB* (Figure 4*C*, Supplementary Figure 4*D*), whereas CsiR could not bind to the DNA fragment of *kana* under the same experimental conditions (Supplementary Figure 4*E*). Chromatin immunoprecipitation (ChIP) qPCR demonstrated that the *ilvB* promoter was enriched 3.38-fold in the CsiR-ChIP samples compared to the mock ChIP samples (Figure 4*D*), with no significant difference observed in the fold enrichment of *rpoS*. These findings indicate that CsiR specifically binds to the *ilvB* promoter region both in vitro and in vivo. Using a dye-based DNase I footprint assay, it was found that CsiR bound to a specific sequence containing a 36-bp motif located at −244 to −209 from the translational start site (Figure 4*E*). These results demonstrate that CsiR increases bacterial BBB penetration by enhancing *ilvB* expression through direct binding to its promoter region.

### *ilvB* Promotes *E coli* K1 Invasion of HBMECs by Activating the FAK/PI3K Signaling Pathway to Induce Actin Cytoskeleton Rearrangements

**Figure 5.**
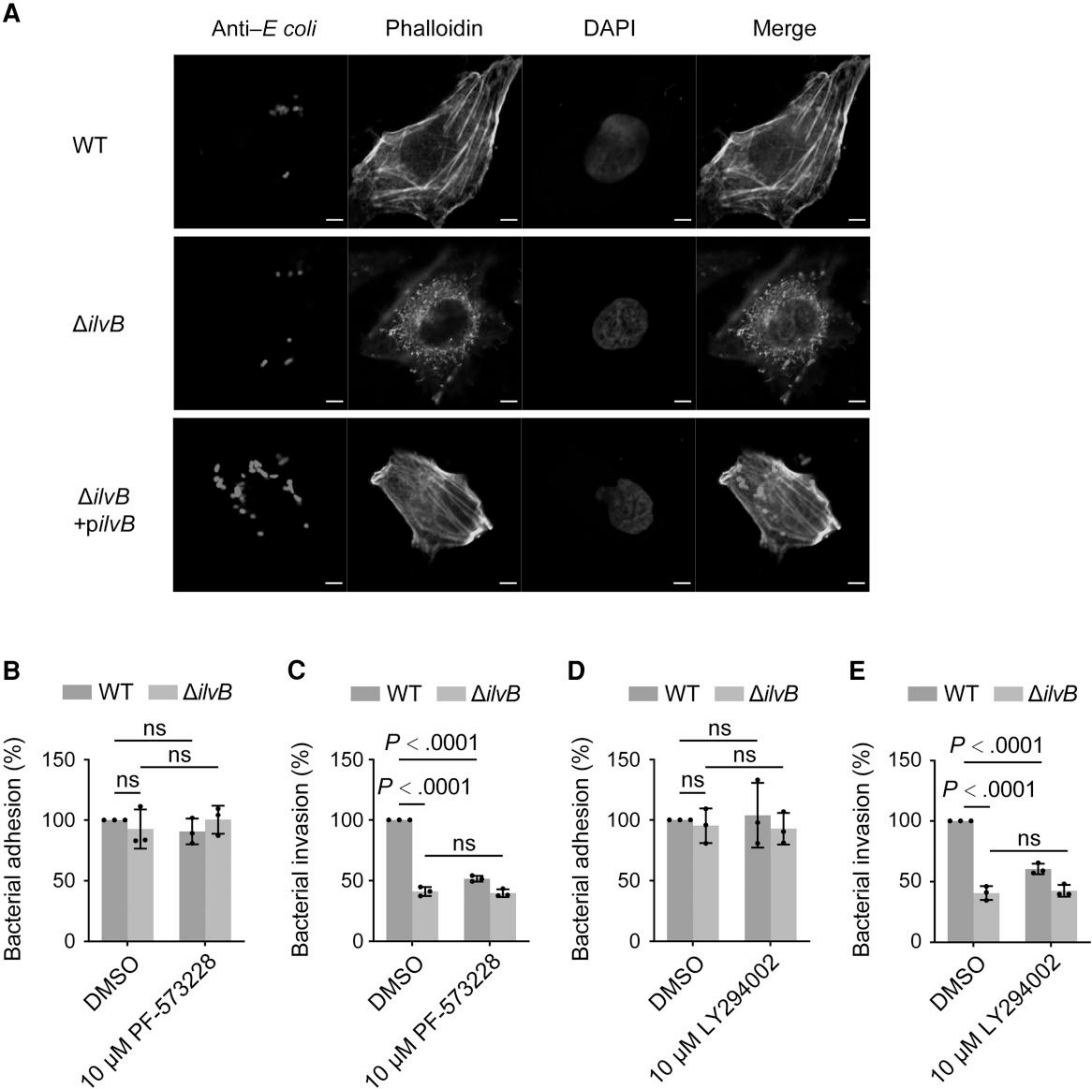
*ilvB* promotes *Escherichia coli* K1 invading human brain microvascular endothelial cells (HBMECs) by activating the FAK/PI3K signaling pathway to induce actin cytoskeleton rearrangements. *A*, Actin cytoskeleton rearrangements in HBMECs induced by wild-type (WT), Δ*ilvB*, and complemented strain. The actin filaments in HBMECs were stained with fluorescein isothiocyanate–phalloidin and visualized by immunofluorescence microscopy. Bar = 5 μm. *B* and *C*, HBMECs were preincubated with 10 μM FAK inhibitor PF573228 or dimethyl sulfoxide (DMSO) for 1 h and infected with WT or Δ*ilvB*. Differences in the adhesion (*B*) and invasion (*C*) of the Δ*ilvB* relative to that of the WT strain were detected in HBMECs. *D* and *E*, HBMECs were preincubated with 10 μM PI3K inhibitor LY294002 or DMSO for 30 min and infected with WT or Δ*ilvB*. Differences in the adhesion (*D*) and invasion (*E*) of the Δ*ilvB* relative to that of the WT strain were detected in HBMECs. Data are presented as mean ± standard deviation (n = 3). Significance is indicated by the *P* values calculated using 2-way analysis of variance; ns, not significant.

The requirement for cytoskeletal rearrangements and activation of FAK and PI3K in *E coli* invasion of HBMECs has been demonstrated [8]. To investigate whether the defects of Δ*ilvB* in cell invasion and transcytosis ability were related to the cytoskeleton rearrangement of HBMECs, we performed an actin filament (F-actin) staining assay to stain the F-actin with fluorescein isothiocyanate–labeled phalloidin at 2.5 hours post infection with infections of WT, Δ*ilvB*, or the complemented strain, respectively. As shown in Figure 5*A*, the WT-infected HBMECs exhibited actin stress fibers extending across the cells, while the Δ*ilvB*-infected HBMECs exhibited actin filaments being accumulated at multiple sites along the cell periphery. Meanwhile, the actin stress fibers observed in the complemented strain-infected HBMECs matched those of the WT-infected HBMECs, indicating that *ilvB* reduces bacterial invasion by interfering with the cytoskeleton rearrangement of HBMECs.

To further examine the impact of FAK on *ilvB*-mediated bacterial invasion, a specific inhibitor was exploited to block the FAK signaling pathway. Inhibition of FAK activity with PF-573228 had no effect on bacterial adherence in either WT or Δ*ilvB* (Figure 5*B*) but resulted in significantly reduced bacterial invasion in WT (but not in Δ*ilvB*) (Figure 5*C*). These results indicate that *ilvB* promotes *E coli* K1 invasion by inducing FAK signaling. Similarly, inhibition of PI3K activity with LY294002, a signal downstream of FAK, had no effect on bacterial adherence in either WT or Δ*ilvB* (Figure 5*D*) but resulted in significantly reduced bacterial invasion in WT (but not in Δ*ilvB*) (Figure 5*E*), indicating that *ilvB* promotes *E coli* K1 invasion by inducing PI3K signaling. Overall, these results show that *ilvB* plays an important role in promoting *E coli* K1 invasion of HBMECs by activating the FAK/PI3K signaling pathway to induce actin cytoskeleton rearrangements.

To determine whether the synthesis of BCAAs influence FAK signaling, we performed the F-actin staining assay in HBMECs infected of Δ*ilvB* with or without 10 mM S-2-acetolactate (S-2-AL, intermediate in the first step of leucine) [25]. The results showed that the Δ*ilvB*-infected HBMECs supplemented with 10 mM S-2-AL exhibited actin stress fibers extending across the cells, similar to that of WT (Supplementary Figure 5*A*). In addition, the results of adhesion and invasion assays showed that S-2-AL significantly increased the invasion ability of *ΔilvB* but with no effect on bacterial adherence (Supplementary Figure 5*B* and 5*C*). Moreover, inhibition of FAK activity had no effect on bacterial adherence in either Δ*ilvB* or Δ*ilvB* with 10 mM S-2-AL (Supplementary Figure 5*B*), but resulted in significantly decreased bacterial invasion rate in Δ*ilvB* with 10 mM S-2-AL (Supplementary Figure 5*C*), indicating that the synthesis of BCAAs induces the FAK signaling.

## DISCUSSION

**Figure 6.**
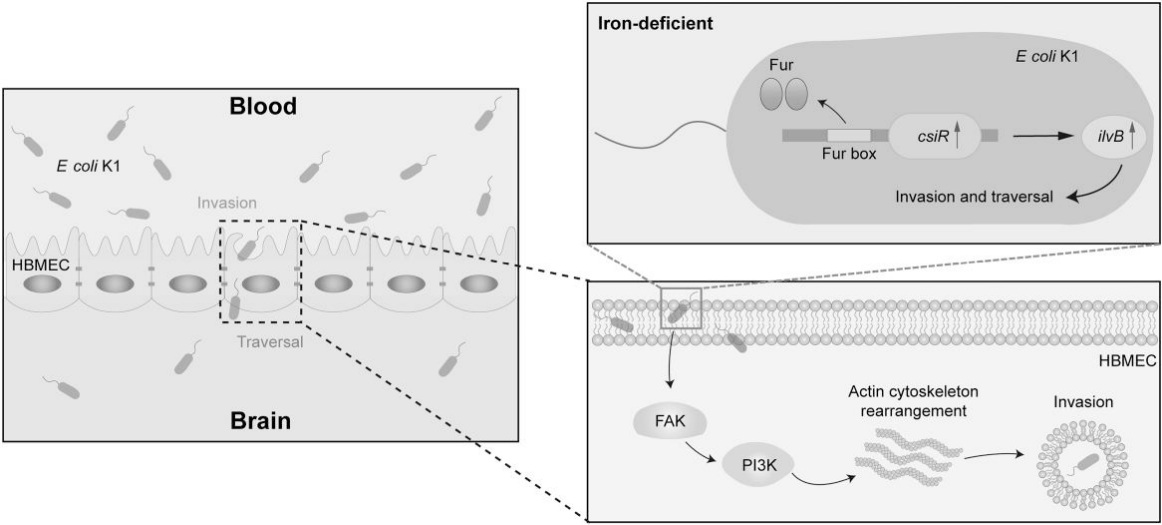
Model of the regulatory pathway of CsiR-mediated signaling in *Escherichia coli* K1. When *E coli* K1 enters the blood, where the iron is scarce, Fur is in an inactive state and relieves the repression of *csiR*, thereby inducing the expression and function of downstream effector IlvB, which activated the FAK/PI3K signaling pathway in human brain microvascular endothelial cells (HBMECs) to induce actin cytoskeleton rearrangements, thus promoting the bacterial invasion and penetration to the blood-brain barrier.

To survive and gain entry into the brain to cause meningitis in the host, *E coli* K1 must overcome 2 major obstacles: nutritional and innate immunity. Nutritional immunity refers to the host's strategy of sequestrating essential nutrients, such as iron, in various storage molecules, or proteins, thereby making them unavailable to pathogens [26]. The host-induced iron limitation conditions can be an accessible signal that pathogens, including *E coli* K1, can use to control their own bacterial virulence. GntR family transcriptional regulators are widespread in bacteria and can activate the transcription of genes to perform multiple functions [17]. However, the roles of the GntR family regulators in *E coli* K1 remain unclear. In this study, a GntR family regulator, CsiR, which promotes bacterial virulence, was characterized in *E coli* K1 (Figure 6). In brief, CsiR is directly controlled by the Fur global regulator in response to the iron concentration through a specific DNA sequence known as the “Fur box” located near the −10 region of the *csiR* promoter. Under iron-sufficient conditions, Fur binds ferrous iron to form an iron-bound Fur, which in turn binds to the Fur box to repress the *csiR* expression. In contrast, when *E coli* K1 enters the blood and HBMECs, where the iron is scarce, Fur is in an inactive state and thus its repression on *csiR* is relieved, thereby inducing the expression and function of downstream effector IlvB, which is regulated by CsiR. Then, IlvB activates the FAK/PI3K signaling pathway to induce actin cytoskeleton rearrangements, thereby enabling bacteria to invade and penetrate the BBB. This not only enhances our understanding of how *E coli* K1 utilizes environmental cues to facilitate bacterial invasion and penetration of the BBB but also provides a novel potential target for the prevention and therapy of *E coli* meningitis.

The *ilvB* gene multivalently controlled by valine and leucine, as well as its expression, is regulated by cyclic adenosine 3′,5′-monophophate (cAMP) [27]. However, our results showed that *ilvB* expression is also in response to the iron signal indirectly by CsiR. Previous studies have shown that BCAAs play an important role in the colonization of invading pathogens in the host [28]. In *Streptococcus pneumoniae*, downregulation of BCAA biosynthesis significantly reduced the bacterial colonization capacity of the host lungs and blood [29]. In *Bacillus anthracis*, both BCAA transport and synthesis are critical for bacterial virulence in a murine model of late-stage anthrax [30]. BCAAs are synthesized in bacteria, plants, and fungi, but not in animals, suggesting that pathogens may encounter BCAA limitations in vivo [31]. BCAAs are small nonpolar amino acids with branched alkyl side chains, which make them hydrophobic, and confer unique properties in proteins. Due to the strong stabilizing effect of Leu on α-helical structures and the preferential presence of Ile and Val in β-sheets [32, 33], *ilvB* mutant-induced BCAA deficiency may disturb the expression and structure of certain proteins critical for *E coli* K1 invasion into HBMECs. However, the precise conclusions for this hypothesis merit further investigation. In *Salmonella enterica* serovar Typhimurium, the activation of the transcription of *ilvB* facilitates BCAA synthesis and thus promotes the recovery of *Salmonella* from nitrosative stress [34]. Nitric oxide (NO) acts as an antimicrobial molecule, and *E coli* upon invasion of HBMECs also produces a higher NO amount by activating inducible NO synthase [35]. The upregulation of *ilvB* upon invasion of HBMECs may also favor *E coli* K1 by resisting the nitrosative stress released by HBMECs, although the precise conclusion needs further research.

For internalization, *E coli* K1 induces actin cytoskeletal rearrangements to trigger a zipper-like mechanism in HBMECs, which engulfs the bacterium into the cell. The host cell signaling molecules are activated in response to specific microbial factors of *E coli* K1 and their interactions with HBMEC factors. These host cell signal transduction pathways include FAK, PI3K, Akt, Src kinase, and Rho GTPases (RhoA and Rac1) [9, 36]. In addition, recent evidence has shown that bacteria exploit fluid phase uptake, or macropinocytic, pathways to gain entry into HBMECs by activating RhoA and PI3K [37]. In this study, we found that *ilvB* induces actin cytoskeleton rearrangements through the FAK/PI3K signaling pathway in the *E coli* K1 infection of HBMECs. As the most abundant of essential amino acids, BCAAs are not only the substrates for synthesis of nitrogenous compounds, they also serve as signaling molecules regulating metabolism of glucose, lipid, and protein synthesis, intestinal health, and immunity [38]. Previous study demonstrated that BCAAs activate mammalian target of rapamycin signaling, which involved in the PI3K-Akt signaling pathway [39]. Therefore, it is likely that *ilvB* induces the FAK/PI3K signaling pathway by promoting the synthesis of BCAAs. However, whether this pathway is also dependent on other factors was not clarified. This highlights the need for further research to elucidate the complete molecular mechanism of *ilvB*-dependent host actin cytoskeleton rearrangements.

## Supplementary Data

Supplementary materials are available at *The Journal of Infectious Diseases* online (http://jid.oxfordjournals.org/). Supplementary materials consist of data provided by the author that are published to benefit the reader. The posted materials are not copyedited. The contents of all supplementary data are the sole responsibility of the authors. Questions or messages regarding errors should be addressed to the author.

## Supplementary Material

jiae157_Supplementary_Data

## Notes

***Author contributions*.** B. L., H. S., and Y. Z. designed the research. Y. Z., Y. W., C. J., X. L., Y. P., and Q. G. performed the research. B. L., H. S., and Y. Z. analyzed the data. B. L., H. S., and Y. Z. wrote the manuscript.

***Data availability*.** The RNA-seq data are available at the Sequence Read Archive database (PRJNA994443). All data are available in the manuscript and the Supplementary Materials.

***Financial support***. This work was funded by the National Natural Science Foundation of China (grant numbers 32070130, 81772148, 31820103002, 31770144, and 81871624); Science Fund for Distinguished Young Scholars of Tianjin Municipality (award number 20JCJQJC00180); Natural Science Foundation of Tianjin Municipality (award number 20JCZDJC00820); and Science and Technology Foundation of Shenzhen City (grant numbers JCYJ20220530164604010, JCYJ20230807151559009).
