# Supplementary material for: CsiR-Mediated Signal Transduction Pathway in Response to Low Iron Conditions Promotes Escherichia coli K1 Invasion and Penetration of the Blood-Brain Barrier
Source: J Infect Dis. 2024 Mar 26;230(4):e807–17. doi: 10.1093/infdis/jiae157 (PMC11481304; doi:10.1093/infdis/jiae157)
Supplement: jiae157_Supplementary_Data [file jiae157_supplementary_data.zip › Supplementary_Figure legends.docx]

Supplementary Figure legends for

**CsiR-mediated signal transduction pathway in response to low iron conditions promotes *Escherichia coli* K1 invasion and penetration of the blood–brain barrier**

Yangyang Zheng^1,2,3*^, Hao Sun^1,2*^ Yanling Wang^1,2^, Chen Jin^1,2^, Xiaoya Li^1,2^, Yu Pang^1,2^, Qianwen Ge^1,2^, Lei Wang^1,2^, and Bin Liu^1,2,4#^

**Supplementary figure 1**

**Figure S1. *csiR* deletion does not affect *E. coli* K1 growth and adhesion of HBMECs.** (a) Growth of WT and Δ*csiR* strains in LB medium. (b) Growth of WT and Δ*csiR* strains in DMEM. (c) Differences in the adhesion of the Δ*csiR* and complemented strain relative to that of the WT. (d) The TEER of HBMEC monolayer infected with WT, Δ*csiR* and complemented strains. The data are represented as the mean ± SD (n = 3). Significance is calculated using the Student's *t* test (c) and one-way ANOVA (d). ns, not significant.

**Supplementary figure 2**

**Figure S2.** (a) Purified Fur protein analysis via SDS-PAGE. (b) EMSA and competition assays of the specific binding of purified Fur to the region of *kana* (negative control). (c) Growth of WT, Δ*fur*, and Fur H118A strains in LB medium. (d) Growth of WT, Δ*fur*, and Fur H118A strains in DMEM. (e) Differences in the adhesion, invasion and transcytosis of the Δ*fur* relative to that of the WT. (f) qRT-PCR expression level of *csiR* in WT and Δ*fur* in HBMECs. (g) Differences in the adhesion, invasion and transcytosis of the Fur H118A relative to that of the WT. (h) The TEER of HBMEC monolayer infected with WT, Δ*fur*, and Fur H118A strains. The data are represented as the mean ± SD (n = 3). Significance is calculated using the Student's *t* test (e-g) and one-way ANOVA (h). ns, not significant.

**Supplementary figure 3**

**Figure S3.** (a) Heatmap of the expression profiles of different genes in WT and Δ*csiR* infected with HBMECs. Z scores of the relative gene expression levels are displayed in the heatmaps (each sample was mixed by three replicates), with red representing higher and blue representing lower abundance. (b) RNA-seq results validation by qRT-PCR.

**Supplementary figure 4**

**Figure S4. *ilvB* deletion does not affect *E. coli* K1 growth and adhesion of HBMECs.** (a) Growth of WT and Δ*ilvB* strains in LB medium. (b) Growth of WT and Δ*ilvB* strains in DMEM. (c) Differences in the adhesion of the Δ*ilvB* and complemented strain relative to that of the WT strain. (d) Purified CsiR protein analysis via SDS-PAGE. (e) EMSA and competition assays of the specific binding of purified CsiR to the region of *kana* (negative control). (f) The TEER of HBMEC monolayer infected with WT, Δ*ilvB* and complemented strains. The data are represented as the mean ± SD (n = 3). Significance is calculated using the Student's *t* test (c) and one-way ANOVA (f). ns, not significant.

**Supplementary figure 5**

**Figure S5.** (a) Actin cytoskeleton rearrangements in HBMECs induced by Δ*ilvB* and Δ*ilvB* supplemented with 10 mM S-2-AL. The actin filaments in HBMECs were stained with FITC-phalloidin and visualized by immunofluorescence microscopy. Bar = 5 μm. (b, c) HBMECs were pre-incubated with 10 μM FAK inhibitor PF573228 or DMSO for 1 h and infected with Δ*ilvB* or Δ*ilvB* supplemented with 10 mM S-2-AL. Differences in the adhesion (b) and invasion (c) of the Δ*ilvB* supplemented with 10 mM S-2-AL relative to that of the Δ*ilvB* were detected in HBMECs. The data are represented as the mean ± SD (n = 3). Significance is indicated by the *P* values calculated using the two-way ANOVA (b, c). ns, not significant.
