# Supplementary material for: CsiR-Mediated Signal Transduction Pathway in Response to Low Iron Conditions Promotes Escherichia coli K1 Invasion and Penetration of the Blood-Brain Barrier
Source: J Infect Dis. 2024 Mar 26;230(4):e807–17. doi: 10.1093/infdis/jiae157 (PMC11481304; doi:10.1093/infdis/jiae157)
Supplement: jiae157_Supplementary_Data [file jiae157_supplementary_data.zip › Supplementary_Methods.docx]

Supplementary Methods for

**CsiR-mediated signal transduction pathway in response to low iron conditions promotes *Escherichia coli* K1 invasion and penetration of the blood–brain barrier**

Yangyang Zheng^1,2,3*^, Hao Sun^1,2*^ Yanling Wang^1,2^, Chen Jin^1,2^, Xiaoya Li^1,2^, Yu Pang^1,2^, Qianwen Ge^1,2^, Lei Wang^1,2^, and Bin Liu^1,2,4#^

**Cell adhesion and invasion assays**

Adhesion and invasion assays were performed as described by Wang et al. with some modifications [1]. For the bacterial adherence assay, after 90-min incubation of HBMECs with bacteria at a multiplicity of infection (MOI) of 100:1, the cells were washed with sterile PBS and lysed using 0.1% Triton X-100. The bacteria were then collected and enumerated. For the bacterial invasion assay, after 90-min incubation of HBMECs with bacteria, the cells were incubated in Dulbecco's modified eagle medium (DMEM) containing 100 μg/mL gentamicin for 60 min to kill the extracellular bacteria, followed by washing with sterile PBS and lysis with 0.1% Triton X-100. The bacteria were then collected and enumerated. Data were presented as relative adhesion or invasion compared to the WT strain, defined as 100%.

**Transwell assay**

An *in vitro* endothelial barrier model of HBMECs was constructed using collagen-coated polycarbonate in 3 μm pore size Transwell membrane inserts, as previously described with minor modification [2]. Briefly, 2 × 10^5^ HBMECs were seeded on Transwell filters and incubated for 5 days to form tight junctions. The bacteria were added to the cell layers on Transwell filters at a MOI of 100:1 and incubated for 90 min. Both apical and basolateral chambers were washed, and extracellular bacteria were killed by 100 μg/mL gentamicin for 60 min, followed by supplement with DMEM medium containing 25 μg/mL trimethoprim and 50 μM rottlerin. After incubation for additional 4 h, 100 μL samples from the apical, basolateral chambers, and HBMECs lysate on the Transwell filter were collected and enumerated. The percentage of bacterial number in the basolateral chamber to total bacterial count (all sections of Transwell chambers containing apical, basolateral and filter) was described as bacterial transcytosis. Data were presented as relative transcytosis compared to the WT strain, defined as 100%.

**Quantitative real-time PCR (qRT-PCR)**

RNA samples were isolated using TRIzol LS reagent (Invitrogen, #15596018) and cDNA was synthesized using the PrimeScript^TM^ RT reagent Kit (Takara, #RR047A). The qRT-PCR was then performed using SYBR^TM^ Green PCR master mix (Applied Biosystems, #4367659) on an ABI 7500 Real-Time PCR system (Applied Biosystems). The 2^−ΔΔCt^ method was employed to calculate the fold change in the target gene relative to the housekeeping gene (16S rRNA). Three independent biological replicates were performed.

**RNA-seq analysis**

WT and Δ*csiR* strains during HBMEC invasion were enriched and collected after gentamicin treatment. The total RNAs were then isolated using TRIzol reagent following the manufacturer’s instructions. The MICROBEnrich Kit (Thermo Fisher Scientific, #AM1901) was used to deplete host-cell RNA. The MICROBExpress Bacterial mRNA Enrichment Kit (Thermo Fisher Scientific, #AM1905) was subsequently employed to deplete bacterial 23S and 16S rRNAs. RNAs were quantified and qualified, and then the libraries were constructed and analyzed by NOVOGENE, Inc. (Tianjin, China). Quality control and filtering of raw sequences were carried out using FastQC1 and an in-house perl program (NGQC, NOVOGENE). The filtered reads were mapped to the reference genome of *E. coli* RS218 (GenBank accession number: CP007149.1). Gene locus expression levels were normalized using fragments per kilobase of exon model per million mapped fragments (FPKM). Differential gene expression was analyzed using the edgeR package with the default parameters p ≤ 0.05 and |log2FoldChange| ≥1.0. RNA-seq data have been submitted to the National Center for Biotechnology Information (NCBI) Sequence Read Archive database under accession number PRJNA994443.

**Electrophoretic mobility shift assay (EMSA)**

The pET28a-*fur* and pET-28a-*csiR* plasmids were transformed into *E. coli* BL21 (DE3), and the proteins harboring an N-terminal 6× His tag were expressed after subjection to induction using 0.1 mM IPTG. The proteins were purified by HiTrap Ni^2+^ chelating column (GE Healthcare). Protein concentrations were determined by the Bradford protein assay (Bio-Rad) using a BSA standard [3], and the proteins were aliquoted and stored at −80℃. The DNA fragments of *csiR* and *ilvB* promoter were amplified with and without 6-FAM-labeled primers from the genomic DNA of WT as a template, and the *kana* fragment was amplified with and without 6-FAM-labeled primers from the pKD4 DNA as a template. The PCR fragments were purified using a SPARKeasy Gel DNA Extraction Kit (Sparkjade, #AE0101-C). In each case, 10 ng of each labeled DNA probe was incubated with increasing concentrations of proteins (0–2 μM) in binding buffer (20 mM Tris-HCl pH 7.4, 50 mM KCl, 1 mM DTT, 100 μg/mL BSA, and 5% glycerol). The Mn^2+^ ions are routinely used in the Fur-binding experiments in place of Fe^2+^ because of its greater redox stability [4]. Accordingly, both binding and electrophoresis buffers containing 0.1 mM MnCl_2_ were used for all Fur-DNA binding experiments. For competition assays, various concentrations of unlabeled DNA fragments (0–500 ng) were added [5]. The reaction mixtures were incubated for 30 min at 30℃. Native 6% (w/v) polyacrylamide gels were used to separate the samples in 0.5× Tris-borate buffer at 4°C and 90 V for 90 min. Then, labeled fragments were visualized using Amersham Imager 600 (GE Healthcare).

**Animal experiment**

All animal studies were conducted according to protocols approved by the Institutional Animal Care Committee of Nankai University (Tianjin, China) and performed under protocol no. IACUC 2016030502. 18-day-old BALB/c mice were purchased from Beijing Vital River Laboratory Animal Technology Co., Ltd. (licensed by Charles River), and housed in specific-pathogen-free (SPF) mouse facilities. We used 18-day-old BALB/c mice for the tail vein injection model [6]. 1 × 10^7^ CFU of *E. coli* K1 strain with or without 10 μM FeSO_4_ in 100 μL sterile PBS were injected intravenously in which dose bacteria develop a high level of bacteremia followed by bacterial traversal of the BBB mimicking the pathogenesis of human meningitis [7]. At 4 h after bacterial inoculation, 100 μL of blood of each mouse was collected and processed for RNA extraction followed by qRT-PCR analyses on specific genes. The 2^−ΔΔCt^ method was employed to calculate the fold change in the target gene relative to the housekeeping gene (16S rRNA). Three independent biological replicates were performed.

**Dye primer-based DNase Ⅰ footprint assay**

A 300-bp fragment of the *ilvB* promoter regions was generated by PCR with 6-FAM-labeled primers. Various amounts of the CsiR protein were added to 40 ng of the 6-FAM-labeled *ilvB* promoter in the band-shift buffer. 0.05 U DNase Ⅰ was added to a 20 μL reaction for 15 min at 30℃. The reaction was stopped by heating at 70℃ for 10 min in the presence of 250 mM EDTA. DNA fragments were purified and eluted in 15 μL distilled water. The samples and the results were analyzed by MAP Biotech Co., Ltd. and a peak scanner, respectively.

**ChIP-qPCR**

Bacterial cultures were grown to midlogarithmic phase (OD_600 nm_, approximately 0.3), harvested, cross-linked, sonicated, and immunoprecipitated. Anti-3×FLAG antibody (Sigma, #F1804) and protein A magnetic beads were used to enrich the protein-DNA complexes. As a negative control, ChIP was performed on another aliquot without antibody addition. After RNA and protein removal by RNase A and proteinase K treatment, the DNA sample was purified with a PCR purification Kit. To measure the enrichment of CisR-binding targets in the ChIP samples, qRT-PCR assays were performed using SYBR green PCR master mix. Relative target levels were calculated using the 2^−ΔΔCt^ method.

**Actin filament (F-actin) staining assay**

An actin filament (F-actin) staining assay was performed as described by Biswas et al [8]. To evaluate actin cytoskeletons, HBMECs were infected with the WT strain, Δ*ilvB* mutant, and *ilvB* complemented strain, respectively. At 2.5 h p.i., the cells were fixed in 4% paraformaldehyde, then permeabilized in 0.2% Triton X-100, and blocked with 5% BSA at room temperature. The fixed cells were incubated with anti-*E. coli* (Abcam, #ab35654) at 4℃ overnight. F-actin and *E. coli* were visualized with FITC-conjugated phalloidin (YEASEN, #40735ES75) for F-actin and AlexaFluor 594-conjuagted secondary antibody for *E. coli*, respectively, under a Zeiss LSM800 confocal microscope.

**References:**

1. Wang X, Maruvada R, Morris AJ, et al. Sphingosine 1-Phosphate activation of EGFR as a novel target for meningitic *Escherichia coli* penetration of the blood-brain barrier. PLoS Pathog **2016**; 12:e1005926.

2. Zhang WG, Khan AN, Kim KJ, Stins M, Kim KS. Transforming growth factor-beta increases *Escherichia coli* K1 adherence, invasion, and transcytosis in human brain microvascular endothelial cells. Cell Tissue Res **2002**; 309:281-6.

3. Bradford MM. A rapid and sensitive method for the quantitation of microgram quantities of protein utilizing the principle of protein-dye binding. Anal Biochem **1976**; 72:248-54.

4. Gao H, Zhou D, Li Y, et al. The iron-responsive Fur regulon in *Yersinia pestis*. J Bacteriol **2008**; 190:3063-75.

5. Liu Y, Xu T, Wang Q, et al. *Vibrio cholerae* senses human enteric alpha-defensin 5 through a CarSR two-component system to promote bacterial pathogenicity. Commun Biol **2022**; 5:559.

6. Sun H, Wan X, Fan Y, et al. Bacteria reduce flagellin synthesis to evade microglia-astrocyte-driven immunity in the brain. Cell Reports **2022**; 40.

7. Cheng Z, Zheng Y, Yang W, et al. Pathogenic bacteria exploit transferrin receptor transcytosis to penetrate the blood-brain barrier. Proc Natl Acad Sci U S A **2023**; 120:e2307899120.

8. Biswas K, Yoshioka K, Asanuma K, et al. Essential role of class II phosphatidylinositol-3-kinase-C2alpha in sphingosine 1-phosphate receptor-1-mediated signaling and migration in endothelial cells. J Biol Chem **2013**; 288:2325-39.
