# Supplementary material for: CsiR-Mediated Signal Transduction Pathway in Response to Low Iron Conditions Promotes Escherichia coli K1 Invasion and Penetration of the Blood-Brain Barrier
Source: J Infect Dis. 2024 Mar 26;230(4):e807–17. doi: 10.1093/infdis/jiae157 (PMC11481304; doi:10.1093/infdis/jiae157)
Supplement: jiae157_Supplementary_Data [file jiae157_supplementary_data.zip › Supplementary_Tables.docx]

Supplementary Tables for

**CsiR-mediated signal transduction pathway in response to low iron conditions promotes *Escherichia coli* K1 invasion and penetration of the blood–brain barrier**

Yangyang Zheng^1,2,3*^, Hao Sun^1,2*^ Yanling Wang^1,2^, Chen Jin^1,2^, Xiaoya Li^1,2^, Yu Pang^1,2^, Qianwen Ge^1,2^, Lei Wang^1,2^, and Bin Liu^1,2,4#^

**Supplementary Table 1**

**Table S1. Bacterial strains and plasmids used in this study.**

|  | Genotype or description | Source |
| --- | --- | --- |
| ***E. coli* K1 Strains** |  |  |
| WT | Wild-type *E. coli* K1 RS218 isolated from the cere-brospinal fluid of a neonate with meningitis^[242]^ | Dr. Kwang Sik Kim from Johns Hopkins University provided |
| ∆*csiR* | WT strain ∆*csiR::cm* | This study |
| ∆*fur* | WT strain ∆*fur::cm* | This study |
| ∆*ilvB* | WT strain ∆*ilvB::cm* | This study |
| 6*His tagged CsiR | *E.coli* BL21 haboring the pET28a-∆*csiR* | This study |
| 6*His tagged Fur | *E.coli* BL21 haboring the plasmid pET28a-∆*fur* | This study |
| 3*Flag tagged CsiR | The ∆*csiR* harboring the plasmid pTrc99A-∆*csiR*-3*Flag | This study |
| ∆*csiR* +p*csiR* | The ∆*csiR* harboring the plasmid pTrc99A-*csiR* | This study |
| ∆*ilvB* +p*ilvB* | The ∆*ilvB* harboring the plasmid pTrc99A-*ilvB* | This study |
| BL21 (DE3) | For expressing protein | TransGen Biotech |
| **Plasmids** |  |  |
| pKD3 | Template plasmid containing the Cm cassette for λ Red recombination; Cm^R^ | Novagen |
| pKD4 | Template plasmid containing the Km cassette for λ Red recombination; Km^R^ | Novagen |
| pSim | Red recombination plasmid; Bla^R^ | Novagen |
| pET28a | T7 expression vector; Km^R^ | Novagen |
| pTrc99A | Expression vector; Ap^R^ | NTCC |
| pET28a-∆*csiR* | pET28a harboring *csiR* gene; Km^R^ | This study |
| pTrc99A-∆*csiR* | pTrc99A harboring *csiR* gene; Ap^R^ | This study |
| pTrc99A-∆*ilvB* | pTrc99A harboring *ilvB* gene; Ap^R^ | This study |
| pTrc99A-∆*ilvB*-3*Flag | pTrc99A harboring 3*Flag-tagged *ilvB* gene; Ap^R^ | This study |

**Supplementary Table 2**

**Table S2. Primers used in this study.**

| Target gene | Strand | Primer sequence (5'-3') |
| --- | --- | --- |
| Primers for gene mutation | | |
| *csiR* | F | CATTCATAATACGCGCTGATAATGATCAGGAGTCACACCGTGTAGGCTGGAGCTGCTTC |
|  | R | ATTTACCCGGGGCAAGTGTTGCGTATTCCTGAAGAGTAGCATATGAATATCCTCCTTAG |
| *fur* | F | AAGCCAACCTGCAGGTTGGCTTTTCTCGTTCAGGCTGGCGTGTAGGCTGGAGCTGCTTC |
|  | R | CTAATGAAGTGAACCGCTTAGTAACAGGACAGATTCCGCCATATGAATATCCTCCTTAG |
| *fur-ON* | P1 | CTGAAGAGCTGCATCTCGACGAAATTCTCAATGCCTG |
| (Fur H118A) | P2 | GTTAGTCAGGCGAATGCCGGCTTTTGCGGCGATTTCACG |
|  | P3 | CGTGAAATCGCCGCAAAAGCCGGCATTCGCCTGACTAAC |
|  | P4 | GCATTTCACCGAATATGACAACCACTCCGACATCCCAAG |
| *ilvB* | F | TTCCAGAATCACGTTATCATGAGTTGTGTTTTGCATGGCGTGTAGGCTGGAGCTGCTTCG |
|  | R | TCCTCCCGGAAAGTCGGCCCAGAAGAAAAGGACTGGAGCCATATGAATATCCTCCTTAG |
| Primers for gene verification | | |
| *csiR* | F | CGAGCAGAAGGAAGCGAAAT |
|  | R | GGAGTGAATACCACGACGAT |
| *fur* | F | CCATCACGTCAGTGCGGAAGA |
|  | R | GGAGTGAATACCACGACGAT |
| *ilvB* | F | TGGTCGGATCGGACTGATTAC |
|  | R | GGAGTGAATACCACGACGAT |
| pTrc99a | F | TGGCTGTGCAGGTCGTAAATC |
|  | R | CCACACTACCATCGGCGCTA |
| Primers for over-expression strain construction | | |
| *csiR* | F | CGGAATTCTGGCTGGTAATCGGTTTTATTACC |
|  | R | CGGGATCCTTAATTGCCAGCCATCGCCTGCTGG |
| *ilvB* | F | CGGGATCCATGGCAAGTTCGGGCACAACATC |
|  | R | CCAAGCTTTTATTCCCCCACCATTTCAGTATTC |
| Primers for protein purification | | |
| *csiR* | F | CGGGATCCATGACCATTACCTCTCTGGA |
|  | R | CCCTCGAGTTAATTGCCAGCCATCGCCT |
| *fur* | F | CGGAATTCATGACTGATAACAATACCG |
|  | R | CCCAAGCTTTTATTTGCCTTCGTGCGCGTGCTCA |
| Primers for qRT-PCR | | |
| *csiR* | F | GTGGCGTCTATGTCAGAGCA |
|  | R | CAGATCCCACTCATCAAGCA |
| *ilvB* | F | AACTGCCTACCACCATGACTT |
|  | R | TCCGCCTGAATTGCCACAT |
| *fliT* | F | TGGCAACGGAAGAACAATGG |
|  | R | CCATAGGCACTTAACACCGATT |
| *nanA* | F | GGTTGCCGATGGTGGTGTA |
|  | R | GATGTTGTAAGTGCTGCCGATA |
| *lipB* | F | TCTTCAGCCTTACGAGCCAAT |
|  | R | AGTTCACGCACACCGAGTT |
| *nagE* | F | TGGATGCTGCTGGTGATGG |
|  | R | CGAGTAATACAGGCGTCAATGG |
| *kefB* | F | CGCTTCTTCACAACGCTTATTC |
|  | R | ACGACCAATCACCTGACCAA |
| *ppdC* | F | CGATGGTGTTGATGGTGATGAT |
|  | R | TCGGTTGACCTGCCAGTTAG |
| *ygcG* | F | GTGATGATGGCGTTCTACTGTT |
|  | R | GTCGGTGTTATCGTTGCTGAA |
| *ygiW* | F | CGCAGCAGTAATCGCAGTAAT |
|  | R | TGATAGTACCGCTGGCATCTT |
| *yhgG* | F | ATTAGCCAGACACTGAACACTC |
|  | R | ACAGCCGTCAGGTTCTTCC |
| 16S | F | GAAAGCGTGGGGAGCAAAC |
|  | R | ACATGCTCCACCGCTTGTG |
| Primers for EMSA-promoter | | |
| *kana* | F | CATACGCTTGATCCGGCTAC |
|  | R | CGGCCATTTTCCACCATGATAT |
| *P_csiR_* | F | GTGTTGGTAGTGATGCTATTCC |
|  | R | CGCGAATAATATCGTTCTTC |
| *P_csiR-mutant_* | P1 | CGTGGAATATGCCAGGGCTGAAAGCCG |
|  | P2 | GGTGTGACTCCTGTAGTAATACAGCGCGTATTATGAATG |
|  | P3 | CATTCATAATACGCGCTGTATTACTACAGGAGTCACACC |
|  | P4 | TATGAAACGCCTGATGACGCAGATCCCA |
| *P_ilvB_* | F | CCCTGTAAAGCTGTGCTTGTAT |
|  | R | GCTCCAGTCCTTTTCTTCTG |
| Primers for ChIP | | |
| *csiR* | F | GCTCTAGAATAATGATCAGGAGTCACACC |
|  | R | CCAAGCTTTTACTATTTATCGTCGTCATCTTTGTAGTCGATATCATGATCTTTATAATCACCGTCATGGTCTTTGTAGTCATTGCCAGCCATCGCCTGCTG |
| Primers for ChIP-qPCR | | |
| *csiR* | F | GTGGCGTCTATGTCAGAGCA |
|  | R | CAGATCCCACTCATCAAGCA |
| rpoS | F | GTTATCGCAGGGAGCCACA |
|  | R | TTTTACCACCAGACGCAAGTTA |

**Supplementary Table 3**

**Table S3. Genes downregulated in Δ*csiR* strain compared to WT that infected HBMECs by RNA-seq analysis.**

| Gene | Δ*csiR* | WT | log2FoldChange | pvalue | padj |
| --- | --- | --- | --- | --- | --- |
| *kpsU* | 1.39E-17 | 15.1315 | -6.96703 | 0.000517 | 0.03776 |
| *gatZ* | 1.39E-17 | 15.1315 | -6.96703 | 0.000517 | 0.03776 |
| *yfcA* | 1.39E-17 | 13.87229 | -6.84255 | 0.000858 | 0.050146 |
| *rbsC* | 1.39E-17 | 12.61308 | -6.7063 | 0.001437 | 0.071967 |
| *G4123-orf04929* | 1.39E-17 | 11.35387 | -6.55583 | 0.002435 | 0.096213 |
| *yedF* | 1.39E-17 | 10.09466 | -6.38782 | 0.007243 | 0.171175 |
| *ulaA* | 1.39E-17 | 10.09466 | -6.38782 | 0.007243 | 0.171175 |
| *G4123-orf05387* | 1.39E-17 | 8.835455 | -6.19764 | 0.012731 | 0.229422 |
| *gabT* | 1.39E-17 | 8.835455 | -6.19764 | 0.012731 | 0.229422 |
| *mglB* | 0.775604 | 57.94455 | -5.98582 | 1.30E-10 | 9.32E-08 |
| *yejE* | 1.39E-17 | 7.576256 | -5.97852 | 0.022677 | 0.306173 |
| *agaW* | 1.39E-17 | 7.576256 | -5.97852 | 0.022677 | 0.306173 |
| *tauC* | 1.39E-17 | 7.576256 | -5.97852 | 0.022677 | 0.306173 |
| *G4123-orf02535* | 1.39E-17 | 7.576256 | -5.97852 | 0.022677 | 0.306173 |
| *G4123-orf01857* | 1.39E-17 | 7.576256 | -5.97852 | 0.022677 | 0.306173 |
| *gadE* | 1.39E-17 | 6.317066 | -5.72005 | 0.040965 | 0.397135 |
| *G4123-orf05132* | 1.39E-17 | 6.317066 | -5.72005 | 0.040965 | 0.397135 |
| *hypE* | 1.39E-17 | 6.317066 | -5.72005 | 0.040965 | 0.397135 |
| *ygbA* | 1.39E-17 | 6.317066 | -5.72005 | 0.040965 | 0.397135 |
| *carB* | 1.39E-17 | 6.317066 | -5.72005 | 0.040965 | 0.397135 |
| *G4123-orf01593* | 1.39E-17 | 6.317066 | -5.72005 | 0.040965 | 0.397135 |
| *G4123-orf05337* | 1.39E-17 | 6.317066 | -5.72005 | 0.040965 | 0.397135 |
| *G4123-orf01869* | 1.39E-17 | 6.317066 | -5.72005 | 0.040965 | 0.397135 |
| *glpT* | 4.746673 | 112.0902 | -4.52095 | 7.67E-12 | 1.10E-08 |
| *efeO* | 0.775604 | 20.16835 | -4.46792 | 4.78E-05 | 0.007347 |
| *fucP* | 0.775604 | 17.64992 | -4.27652 | 0.00012 | 0.014416 |
| *G4123-orf05583* | 0.775604 | 16.39071 | -4.17036 | 0.000315 | 0.030169 |
| *melR* | 3.158212 | 55.42615 | -4.07335 | 2.30E-08 | 1.10E-05 |
| *sinH* | 0.775604 | 15.1315 | -4.05577 | 0.000517 | 0.03776 |
| *nikA* | 0.775604 | 12.61308 | -3.79504 | 0.001437 | 0.071967 |
| *nanA* | 30.16026 | 384.0774 | -3.6644 | 3.57E-13 | 1.54E-09 |
| *galS* | 2.363955 | 28.98282 | -3.53776 | 5.75E-05 | 0.008129 |
| *rbsD* | 31.74859 | 366.4486 | -3.52292 | 1.43E-12 | 3.08E-09 |
| *int* | 0.775604 | 10.09466 | -3.47656 | 0.021567 | 0.306173 |
| *yjcU* | 0.775604 | 10.09466 | -3.47656 | 0.021567 | 0.306173 |
| *srlA* | 0.775604 | 10.09466 | -3.47656 | 0.021567 | 0.306173 |
| *caiB* | 1.569683 | 17.64992 | -3.37574 | 0.000885 | 0.050146 |
| *cdd* | 31.74859 | 316.0806 | -3.30966 | 2.57E-11 | 2.76E-08 |
| *csiR* | 1.569683 | 15.1315 | -3.155 | 0.003346 | 0.110509 |
| *ybdH* | 12.68857 | 108.3126 | -3.07914 | 7.11E-08 | 3.06E-05 |
| *ynfE* | 3.158212 | 26.4644 | -3.0097 | 0.000589 | 0.039638 |
| *ibpB* | 350.2053 | 2611.601 | -2.89814 | 8.15E-11 | 7.02E-08 |
| *udp* | 134.9891 | 988.4929 | -2.87105 | 2.71E-10 | 1.67E-07 |
| *rbsA* | 12.68857 | 93.20222 | -2.86259 | 6.73E-07 | 0.000223 |
| *asnA* | 2.363955 | 17.64992 | -2.82543 | 0.003515 | 0.110509 |
| *oxc* | 3.158212 | 22.68677 | -2.78841 | 0.001665 | 0.078885 |
| *entD* | 1.569683 | 11.35387 | -2.7438 | 0.013412 | 0.234556 |
| *yjgK* | 1.569683 | 11.35387 | -2.7438 | 0.013412 | 0.234556 |
| *ebgA* | 16.65944 | 109.5718 | -2.70675 | 8.02E-07 | 0.000234 |
| *dacD* | 3.158212 | 21.42756 | -2.7064 | 0.00237 | 0.096213 |
| *G4123-orf05611* | 3.158212 | 21.42756 | -2.7064 | 0.00237 | 0.096213 |
| *G4123-orf03496* | 11.10022 | 70.53658 | -2.65179 | 6.48E-06 | 0.001268 |
| *ompW* | 8.717668 | 55.42615 | -2.64821 | 2.74E-05 | 0.004919 |
| *soxS* | 106.3995 | 663.6196 | -2.6392 | 6.58E-09 | 3.54E-06 |
| *phnC* | 1.569683 | 10.09466 | -2.57579 | 0.021567 | 0.306173 |
| *aapP* | 1.569683 | 10.09466 | -2.57579 | 0.021567 | 0.306173 |
| *chpR* | 3.95245 | 22.68677 | -2.47717 | 0.001665 | 0.078885 |
| *ygaU* | 3.158212 | 17.64992 | -2.42804 | 0.010034 | 0.203845 |
| *speF* | 3.158212 | 17.64992 | -2.42804 | 0.010034 | 0.203845 |
| *cirA* | 23.01279 | 122.1638 | -2.40078 | 4.21E-06 | 0.000906 |
| *ynfD* | 3.95245 | 21.42756 | -2.39516 | 0.00237 | 0.096213 |
| *G4123-orf03462* | 2.363955 | 12.61308 | -2.34396 | 0.027043 | 0.339731 |
| *yliC* | 2.363955 | 12.61308 | -2.34396 | 0.027043 | 0.339731 |
| *G4123-orf02550* | 2.363955 | 12.61308 | -2.34396 | 0.027043 | 0.339731 |
| *G4123-orf04077* | 6.335088 | 32.76045 | -2.34342 | 0.001153 | 0.062846 |
| *metF* | 43.661 | 221.6406 | -2.33986 | 9.49E-07 | 0.000255 |
| *ibpA* | 363.706 | 1818.305 | -2.32128 | 1.05E-07 | 4.10E-05 |
| *yebE* | 34.92524 | 175.0502 | -2.32051 | 2.64E-06 | 0.000632 |
| *ydbK* | 5.540884 | 27.72361 | -2.29216 | 0.002142 | 0.096116 |
| *htpG* | 352.5878 | 1652.091 | -2.22776 | 3.12E-07 | 0.000112 |
| *yohJ* | 3.95245 | 18.90914 | -2.21567 | 0.016493 | 0.269071 |
| *uhpT* | 3.158212 | 15.1315 | -2.20729 | 0.030348 | 0.369236 |
| *fliT* | 3.158212 | 15.1315 | -2.20729 | 0.030348 | 0.369236 |
| *glpA* | 36.51356 | 168.7542 | -2.20378 | 6.08E-06 | 0.001247 |
| *yqcE* | 2.363955 | 11.35387 | -2.19349 | 0.041032 | 0.397135 |
| *asr* | 6.335088 | 28.98282 | -2.16724 | 0.00349 | 0.110509 |
| *rpmE* | 974.4117 | 4225.894 | -2.11648 | 8.13E-07 | 0.000234 |
| *G4123-orf00901* | 3.158212 | 13.87229 | -2.08281 | 0.043991 | 0.414003 |
| *G4123-orf05984* | 3.158212 | 13.87229 | -2.08281 | 0.043991 | 0.414003 |
| *ndh* | 196.9333 | 817.2419 | -2.05221 | 3.02E-06 | 0.000685 |
| *nagE* | 23.80695 | 98.23902 | -2.03796 | 0.000101 | 0.012745 |
| *moeB* | 15.0711 | 61.72216 | -2.02304 | 0.000403 | 0.034715 |
| *ybgE* | 9.511853 | 37.79727 | -1.97327 | 0.002551 | 0.098985 |
| *deoC* | 30.95443 | 120.9046 | -1.96037 | 0.000104 | 0.012745 |
| *metI* | 45.24932 | 172.5318 | -1.9273 | 5.63E-05 | 0.008129 |
| *yhdW* | 7.923479 | 30.24203 | -1.9119 | 0.005291 | 0.14242 |
| *yijF* | 7.923479 | 30.24203 | -1.9119 | 0.005291 | 0.14242 |
| *G4123-orf01926* | 3.95245 | 15.1315 | -1.89605 | 0.045202 | 0.414003 |
| *ygcU* | 3.95245 | 15.1315 | -1.89605 | 0.045202 | 0.414003 |
| *ybgT* | 6.335088 | 23.94598 | -1.89288 | 0.01438 | 0.243841 |
| *sdaB* | 8.717668 | 32.76045 | -1.89141 | 0.004613 | 0.132003 |
| *marA* | 28.57194 | 105.7942 | -1.88293 | 0.000268 | 0.027454 |
| *yfhD* | 11.8944 | 42.8341 | -1.83501 | 0.002618 | 0.10067 |
| *ycfR* | 27.77778 | 99.49822 | -1.83497 | 0.00038 | 0.034715 |
| *waaF* | 12.68857 | 45.35251 | -1.82505 | 0.00291 | 0.105456 |
| *ycjF* | 9.511853 | 34.01965 | -1.82178 | 0.006485 | 0.163345 |
| *yajO* | 11.10022 | 39.05648 | -1.80063 | 0.003605 | 0.11176 |
| *glpF* | 13.48275 | 46.61172 | -1.77782 | 0.003196 | 0.110129 |
| *flhD* | 9.511853 | 32.76045 | -1.76753 | 0.008204 | 0.182143 |
| *dctA* | 9.511853 | 32.76045 | -1.76753 | 0.008204 | 0.182143 |
| *aspA* | 161.9904 | 551.5508 | -1.76661 | 5.85E-05 | 0.008129 |
| *yecD* | 4.746673 | 16.39071 | -1.75478 | 0.032292 | 0.383706 |
| *G4123-orf06430* | 5.540884 | 18.90914 | -1.74256 | 0.033376 | 0.384203 |
| *nrdB* | 5.540884 | 18.90914 | -1.74256 | 0.033376 | 0.384203 |
| *hipB* | 7.129286 | 23.94598 | -1.72597 | 0.026105 | 0.336634 |
| *csgG* | 7.129286 | 23.94598 | -1.72597 | 0.026105 | 0.336634 |
| *rplA* | 3658.658 | 12058.11 | -1.72057 | 4.61E-05 | 0.007347 |
| *tuf2* | 38.89604 | 128.4598 | -1.71959 | 0.000469 | 0.037387 |
| *napF* | 19.83612 | 65.49977 | -1.71546 | 0.00192 | 0.088911 |
| *yfeY* | 35.7194 | 117.127 | -1.70891 | 0.000602 | 0.039874 |
| *tdcA* | 19.83612 | 64.24057 | -1.68749 | 0.002232 | 0.096213 |
| *galP* | 23.80695 | 76.8326 | -1.68379 | 0.00149 | 0.072946 |
| *fba* | 46.04348 | 147.3478 | -1.67478 | 0.000488 | 0.03776 |
| *sdaC* | 87.3397 | 277.0454 | -1.66364 | 0.00024 | 0.025264 |
| *ycjX* | 76.2215 | 241.7878 | -1.66344 | 0.000278 | 0.027891 |
| *srlR* | 16.65944 | 52.90774 | -1.65784 | 0.004015 | 0.122646 |
| *yrfH* | 115.1352 | 362.671 | -1.65399 | 0.000207 | 0.022808 |
| *tusB* | 65.10329 | 204.0118 | -1.64548 | 0.0004 | 0.034715 |
| *sucA* | 19.04195 | 59.20376 | -1.62842 | 0.003301 | 0.110509 |
| *yhbH* | 18.24778 | 56.68535 | -1.62682 | 0.004523 | 0.132003 |
| *sucB* | 14.27692 | 44.0933 | -1.61612 | 0.008735 | 0.188106 |
| *purD* | 110.3702 | 336.2278 | -1.6057 | 0.000311 | 0.030169 |
| *idhA* | 55.57339 | 168.7542 | -1.5997 | 0.000767 | 0.047866 |
| *yhiP* | 18.24778 | 55.42615 | -1.59446 | 0.006203 | 0.159279 |
| *G4123-orf04453* | 9.511853 | 28.98282 | -1.59135 | 0.021096 | 0.306173 |
| *yebK* | 23.80695 | 71.79579 | -1.5861 | 0.002894 | 0.105456 |
| *G4123-orf00006* | 7.923479 | 23.94598 | -1.57639 | 0.026105 | 0.336634 |
| *yncJ* | 14.27692 | 42.8341 | -1.57441 | 0.010512 | 0.2093 |
| *rplK* | 2665.169 | 7909.051 | -1.56922 | 0.000192 | 0.02175 |
| *metN* | 136.5774 | 405.4837 | -1.56881 | 0.000397 | 0.034715 |
| *degP* | 442.3274 | 1310.848 | -1.56697 | 0.000239 | 0.025264 |
| *aceF* | 244.5826 | 708.9507 | -1.53475 | 0.000392 | 0.034715 |
| *ybiH* | 18.24778 | 52.90774 | -1.5275 | 0.008516 | 0.185238 |
| *ycfS* | 56.36755 | 162.4582 | -1.52446 | 0.001151 | 0.062846 |
| *ybeD* | 262.054 | 753.0227 | -1.52226 | 0.000415 | 0.035076 |
| *nupC* | 63.51497 | 182.6054 | -1.52119 | 0.001195 | 0.064335 |
| *ybgK* | 8.717668 | 25.20519 | -1.5145 | 0.034035 | 0.384203 |
| *manX* | 38.10188 | 108.3126 | -1.50334 | 0.002318 | 0.096213 |
| *glnA* | 398.6488 | 1125.746 | -1.49732 | 0.000457 | 0.037147 |
| *dmsC* | 11.10022 | 31.50124 | -1.49137 | 0.026832 | 0.339731 |
| *glnH* | 107.1936 | 300.9702 | -1.48801 | 0.000883 | 0.050146 |
| *ilvC* | 14.27692 | 40.31569 | -1.4872 | 0.018332 | 0.28922 |
| *dmsA* | 10.30604 | 28.98282 | -1.47727 | 0.033533 | 0.384203 |
| *yfeK* | 13.48275 | 37.79727 | -1.47613 | 0.018121 | 0.28694 |
| *ybhQ* | 28.57194 | 79.351 | -1.46845 | 0.005228 | 0.14242 |
| *yjfN* | 15.86527 | 44.0933 | -1.46533 | 0.012963 | 0.232632 |
| *edd* | 9.511853 | 26.4644 | -1.46068 | 0.033832 | 0.384203 |
| *nhaA* | 790.1676 | 2172.14 | -1.4587 | 0.000553 | 0.039638 |
| *ntpA* | 20.63029 | 56.68535 | -1.45104 | 0.009031 | 0.191166 |
| *neuB* | 52.39676 | 142.311 | -1.43868 | 0.002774 | 0.104809 |
| *nupG* | 19.04195 | 51.64853 | -1.43181 | 0.00998 | 0.203845 |
| *yeiB* | 23.80695 | 64.24057 | -1.42593 | 0.00847 | 0.185182 |
| *G4123-orf05695* | 41.27852 | 110.831 | -1.42134 | 0.003404 | 0.110509 |
| *kpsT* | 26.98361 | 71.79579 | -1.40639 | 0.007069 | 0.171175 |
| *rpsF* | 841.7877 | 2228.804 | -1.40457 | 0.000872 | 0.050146 |
| *metB* | 30.95443 | 81.8694 | -1.39846 | 0.006213 | 0.159279 |
| *rpsP* | 705.1929 | 1839.711 | -1.38318 | 0.001055 | 0.058987 |
| *ycfJ* | 26.98361 | 70.53658 | -1.3809 | 0.009055 | 0.191166 |
| *ygiM* | 84.95723 | 220.3814 | -1.37349 | 0.002548 | 0.098985 |
| *fhuA* | 123.0767 | 318.599 | -1.37101 | 0.002002 | 0.091739 |
| *lipB* | 72.25071 | 186.383 | -1.36519 | 0.002982 | 0.105456 |
| *pflA* | 70.66239 | 181.3462 | -1.35769 | 0.00346 | 0.110509 |
| *hslV* | 231.8761 | 594.3636 | -1.35737 | 0.001667 | 0.078885 |
| *ilvB* | 68.27992 | 173.791 | -1.34571 | 0.00346 | 0.110509 |
| *G4123-orf06269* | 14.27692 | 36.53807 | -1.34564 | 0.031942 | 0.383219 |
| *yeeI* | 14.27692 | 36.53807 | -1.34564 | 0.031942 | 0.383219 |
| *yjiA* | 18.24778 | 46.61172 | -1.34508 | 0.022074 | 0.306173 |
| *rplY* | 1716.153 | 4344.258 | -1.33985 | 0.001385 | 0.071967 |
| *metQ* | 173.9027 | 438.2229 | -1.33256 | 0.002248 | 0.096213 |
| *cpxP* | 2147.38 | 5403.245 | -1.33118 | 0.001471 | 0.072814 |
| *carB* | 13.48275 | 34.01965 | -1.32464 | 0.039238 | 0.397135 |
| *ybdL* | 19.04195 | 47.87092 | -1.32246 | 0.018835 | 0.293926 |
| *hslU* | 1018.09 | 2536.049 | -1.31658 | 0.001721 | 0.080581 |
| *ybfM* | 29.3661 | 73.05499 | -1.30998 | 0.013481 | 0.234556 |
| *pck* | 493.1534 | 1211.371 | -1.29625 | 0.002207 | 0.096213 |
| *rpsR* | 1936.134 | 4729.573 | -1.28846 | 0.00207 | 0.093834 |
| *frdA* | 49.22012 | 119.6454 | -1.27858 | 0.007273 | 0.171175 |
| *sitA* | 18.24778 | 44.0933 | -1.26513 | 0.035482 | 0.390718 |
| *cdaR* | 44.45516 | 107.0534 | -1.26474 | 0.010383 | 0.208975 |
| *ydhC* | 24.60112 | 59.20376 | -1.26125 | 0.021708 | 0.306173 |
| *prlC* | 130.2242 | 309.7846 | -1.24919 | 0.004567 | 0.132003 |
| *uxuR* | 38.10188 | 90.68382 | -1.24731 | 0.011675 | 0.224481 |
| *rpsL* | 2201.382 | 5220.661 | -1.24576 | 0.002863 | 0.105456 |
| *ybeJ* | 111.9586 | 265.7126 | -1.24565 | 0.00535 | 0.143128 |
| *G4123-orf04507* | 15.86527 | 37.79727 | -1.2436 | 0.037621 | 0.397135 |
| *yciV* | 57.95587 | 137.2742 | -1.24162 | 0.008681 | 0.187876 |
| *upp* | 183.4326 | 433.1861 | -1.23898 | 0.0045 | 0.132003 |
| *fusA* | 9732.36 | 22962.78 | -1.23842 | 0.002948 | 0.105456 |
| *ubiX* | 44.45516 | 104.535 | -1.23043 | 0.013338 | 0.234556 |
| *hslO* | 237.4352 | 554.0692 | -1.22195 | 0.004628 | 0.132003 |
| *cydD* | 16.65944 | 39.05648 | -1.22089 | 0.043684 | 0.414003 |
| *purB* | 55.57339 | 129.719 | -1.22043 | 0.010492 | 0.2093 |
| *yjjG* | 28.57194 | 66.75898 | -1.21951 | 0.021394 | 0.306173 |
| *cydB1* | 57.95587 | 133.4966 | -1.2014 | 0.011378 | 0.221007 |
| *tsf* | 2618.314 | 6018.993 | -1.20083 | 0.003991 | 0.122646 |
| *neuS* | 47.6318 | 109.5718 | -1.19899 | 0.013506 | 0.234556 |
| *focA* | 62.72082 | 142.311 | -1.17985 | 0.012431 | 0.229422 |
| *ppx* | 20.63029 | 46.61172 | -1.16931 | 0.039664 | 0.397135 |
| *glmS* | 133.4008 | 299.711 | -1.16678 | 0.008433 | 0.185182 |
| *yjiY* | 34.13108 | 76.8326 | -1.16664 | 0.025639 | 0.335647 |
| *gapA* | 767.9312 | 1722.606 | -1.16537 | 0.005559 | 0.146882 |
| *dnaK* | 1987.754 | 4441.217 | -1.15975 | 0.005437 | 0.14454 |
| *thiJ* | 28.57194 | 62.98137 | -1.1356 | 0.034165 | 0.384203 |
| *lon* | 611.4826 | 1342.328 | -1.13413 | 0.007142 | 0.171175 |
| *fxsA* | 350.2053 | 768.1331 | -1.13277 | 0.007699 | 0.177335 |
| *secA* | 122.2826 | 268.231 | -1.13216 | 0.010545 | 0.2093 |
| *hflD* | 73.04487 | 158.6806 | -1.11744 | 0.01518 | 0.254395 |
| *yebC* | 39.6902 | 85.64701 | -1.10625 | 0.027055 | 0.339731 |
| *groES* | 288.2612 | 619.5476 | -1.10338 | 0.009816 | 0.203845 |
| *alx* | 451.0631 | 962.0498 | -1.09249 | 0.009764 | 0.203845 |
| *surA* | 130.2242 | 275.7862 | -1.08154 | 0.014072 | 0.240506 |
| *cydA* | 374.8242 | 785.7619 | -1.06753 | 0.011891 | 0.224623 |
| *neuE* | 26.98361 | 56.68535 | -1.06601 | 0.045117 | 0.414003 |
| *G4123-orf05696* | 57.95587 | 120.9046 | -1.05858 | 0.025596 | 0.335647 |
| *phoB* | 38.10188 | 79.351 | -1.05494 | 0.04341 | 0.414003 |
| *zntA* | 72.25071 | 149.8662 | -1.05078 | 0.025001 | 0.333367 |
| *tsx* | 344.6462 | 713.9875 | -1.0504 | 0.013597 | 0.235188 |
| *yrfG* | 115.1352 | 238.0102 | -1.04656 | 0.018641 | 0.291946 |
| *cspH* | 80.19228 | 164.9766 | -1.0391 | 0.025334 | 0.334709 |
| *glnQ* | 53.98507 | 110.831 | -1.03532 | 0.030316 | 0.369236 |
| *groEL* | 2361.007 | 4822.754 | -1.0304 | 0.013233 | 0.234556 |
| *yfcE* | 60.33834 | 123.423 | -1.03031 | 0.032525 | 0.383706 |
| *yajG* | 65.89745 | 134.7558 | -1.03008 | 0.027593 | 0.344471 |
| *rpsB* | 8395.002 | 16940.03 | -1.01282 | 0.014585 | 0.245382 |
| *clpB* | 316.8508 | 634.658 | -1.00177 | 0.018638 | 0.291946 |
| *rpmF* | 1821.776 | 3647.921 | -1.00166 | 0.016093 | 0.264546 |
