# Supplementary figures and images for: CsiR-Mediated Signal Transduction Pathway in Response to Low Iron Conditions Promotes Escherichia coli K1 Invasion and Penetration of the Blood-Brain Barrier
Source: J Infect Dis. 2024 Mar 26;230(4):e807–17. doi: 10.1093/infdis/jiae157 (PMC11481304; doi:10.1093/infdis/jiae157)

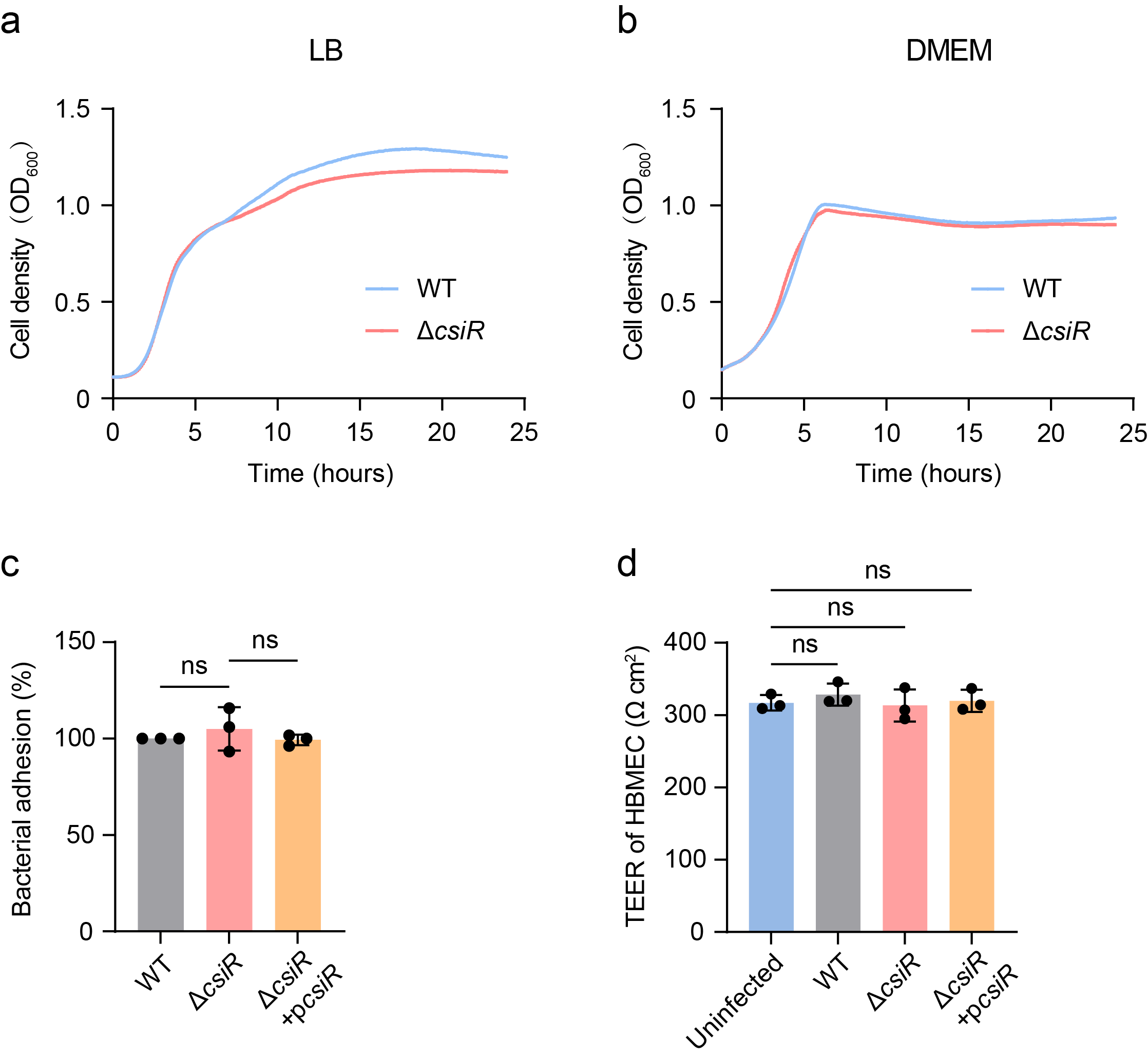

Supplement: jiae157_Supplementary_Data [file jiae157_supplementary_data.zip › S1.png]

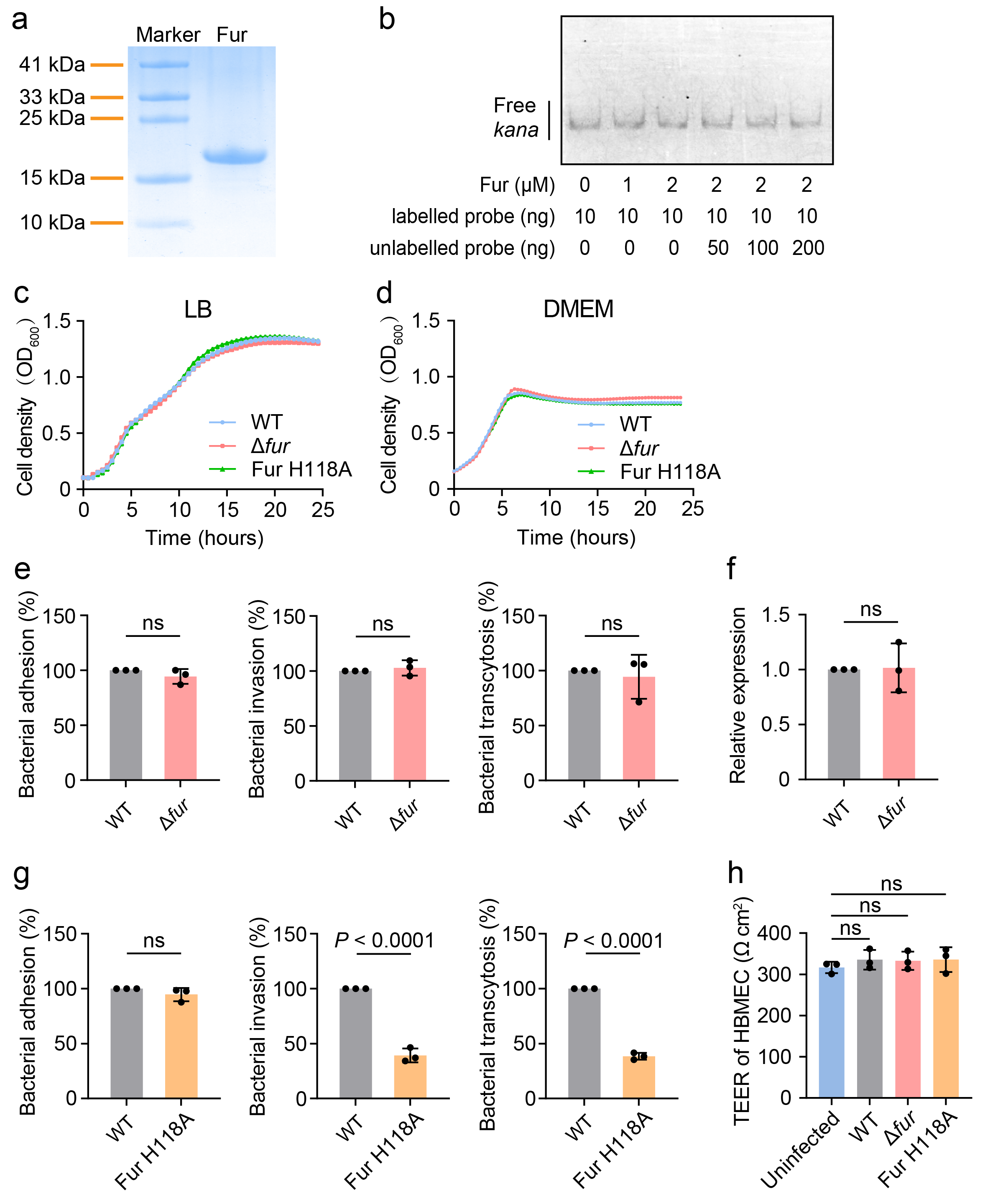

Supplement: jiae157_Supplementary_Data [file jiae157_supplementary_data.zip › S2.png]

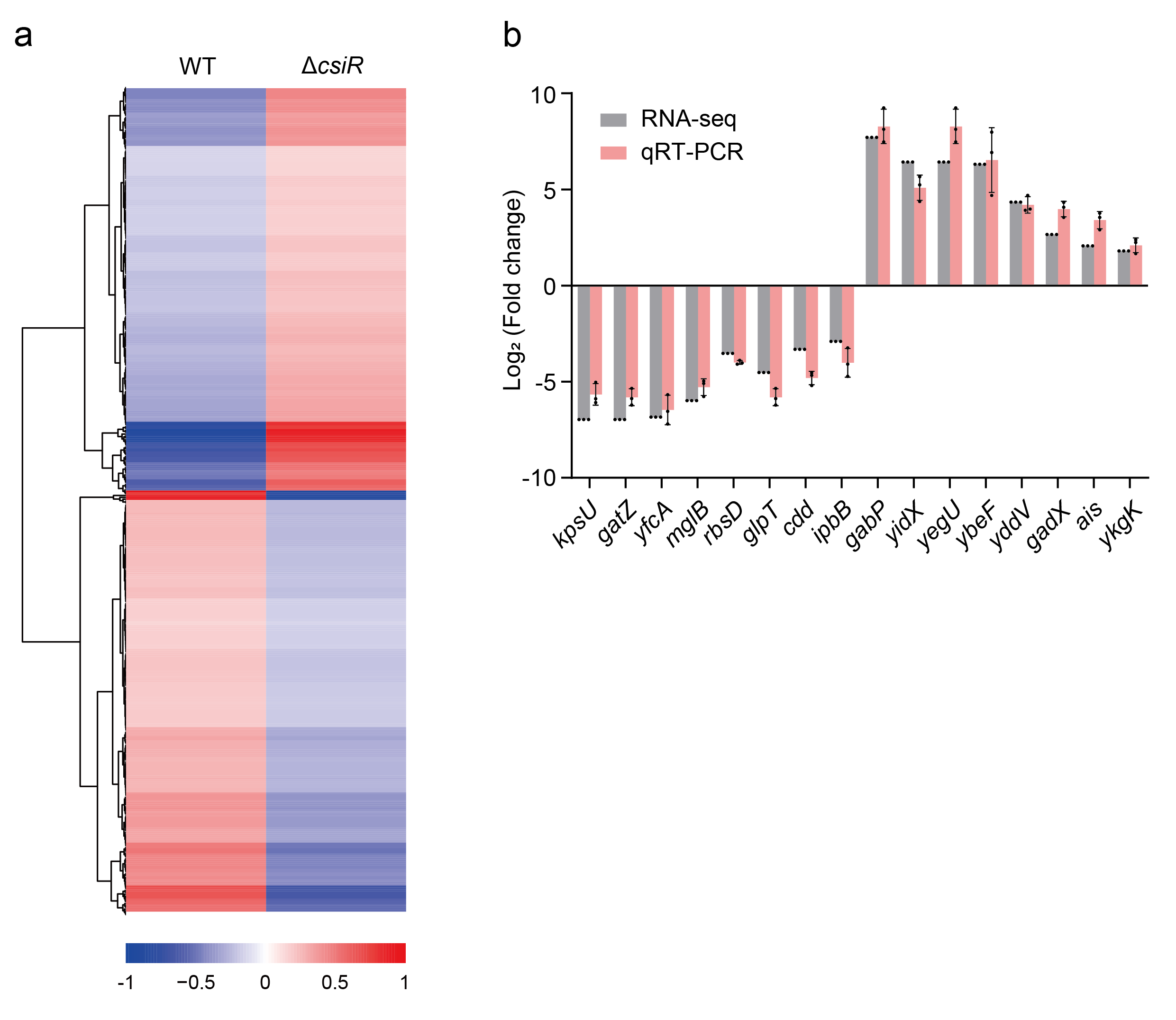

Supplement: jiae157_Supplementary_Data [file jiae157_supplementary_data.zip › S3.png]

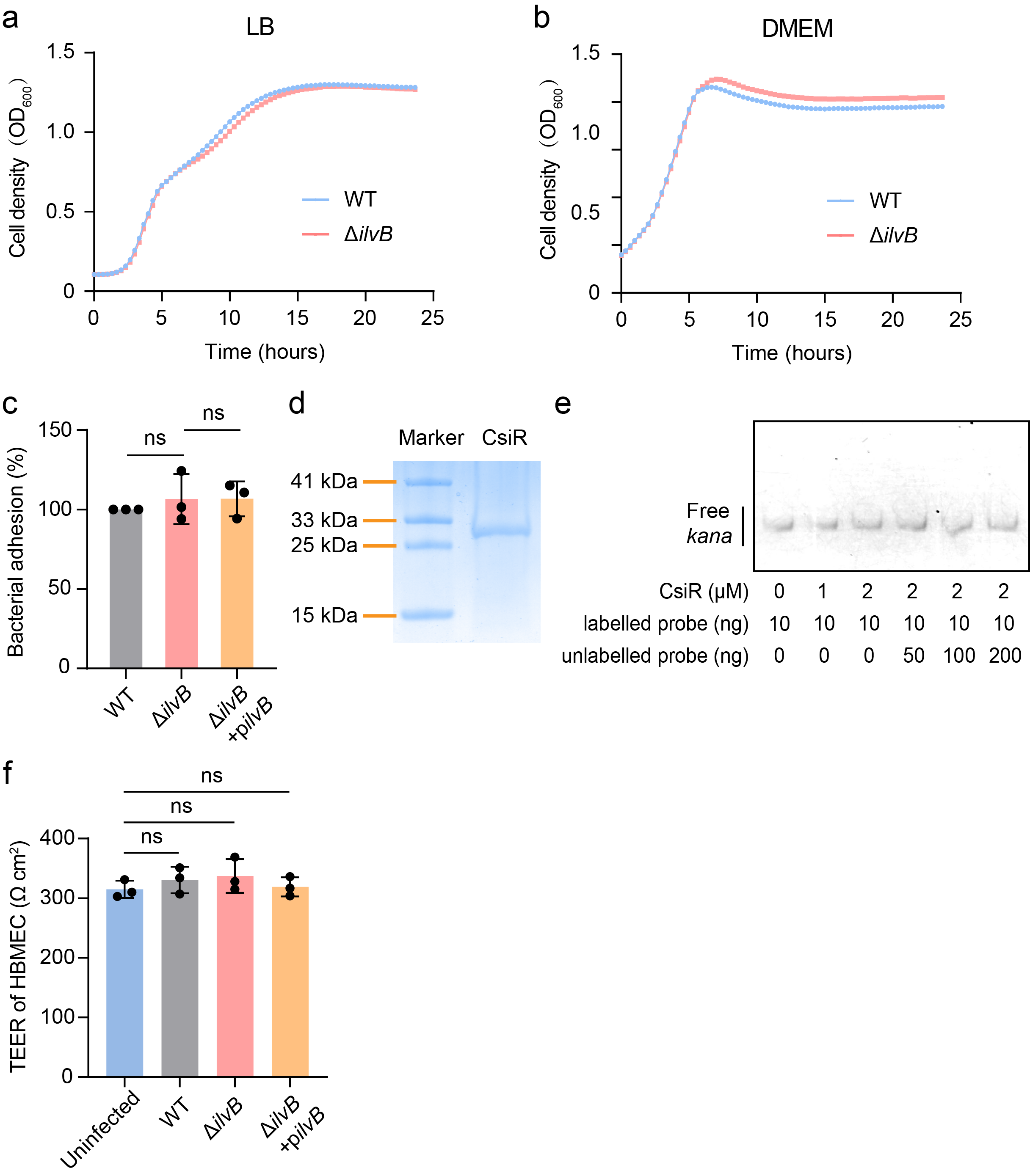

Supplement: jiae157_Supplementary_Data [file jiae157_supplementary_data.zip › S4.png]

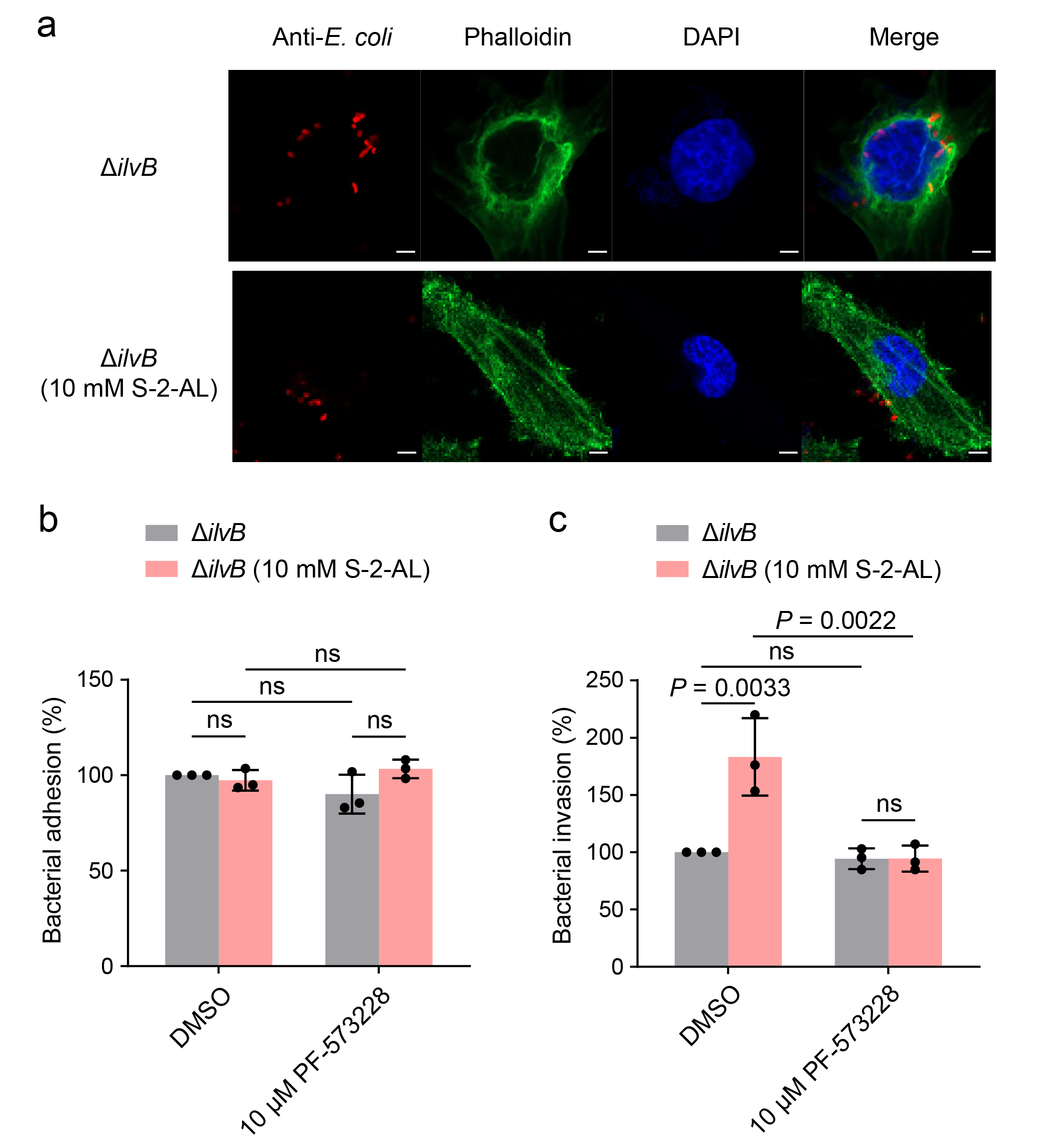

Supplement: jiae157_Supplementary_Data [file jiae157_supplementary_data.zip › S5.png]
